# Butyrate causes loss of specific acetylation by the histone acetyltransferase p300

**DOI:** 10.1016/j.isci.2026.116528

**Published:** 2026-07-08

**Authors:** Muwei Jiang, Anthi Psoma, Jinxiao Lyu, Maria Gabriella Chiariello, Rutger Modderman, Frans Bianchi, Danny Incarnato, Siewert-Jan Marrink, Iris H. Jonkers, Geert van den Bogaart

**Affiliations:** 1 Department of Molecular Immunology, Groningen Biomolecular Sciences and Biotechnology Institute, University of Groningen, 9747AG, Nijenborgh 7, Groningen, the Netherlands; 2 Department of Molecular Dynamics, Groningen Biomolecular Sciences and Biotechnology Institute, University of Groningen, 9747AG, Nijenborgh 7, Groningen, the Netherlands; 3 Department of Genetics, University of Groningen, University Medical Center Groningen, Groningen, the Netherlands; 4 Department of Molecular Genetics, Groningen Biomolecular Sciences and Biotechnology Institute, University of Groningen, 9747AG, Nijenborgh 7, Groningen, the Netherlands

**Keywords:** butyrate, SCFA, p300, acetylation, histones

## Abstract

Butyrate, a short-chain fatty acid produced by microbial fermentation of dietary fiber, exerts beneficial metabolic and immunomodulatory functions through butyrylation of the histone acetyltransferase p300. This is thought to result in histone hyperacetylation at transcription starting sites and enhancers that regulate specific genes. However, we show that butyrate-induced hyperacetylation is not specific but occurs at histones throughout the entire genome. Mechanistically, our data show that butyrylation of p300 prevents its recruitment to acetylated histones through its bromodomain, and thereby p300 cannot maintain the acetylation of specific histones through positive feedback. Thus, the epigenetic regulation of specific genes by butyrate is limited, but butyrate instead increases histone acetylation globally along the entire chromatin structure.

## Highlights

• Activated p300 loses bromodomain binding to acetylated histones
• Butyrylated p300 drives random histone hyperacetylation
• Butyrate lowers site-specific histone acetylation at transcription start sites
• Butyrate increases global histone acetylation across chromatin

## Graphical abstract

**Figure:**
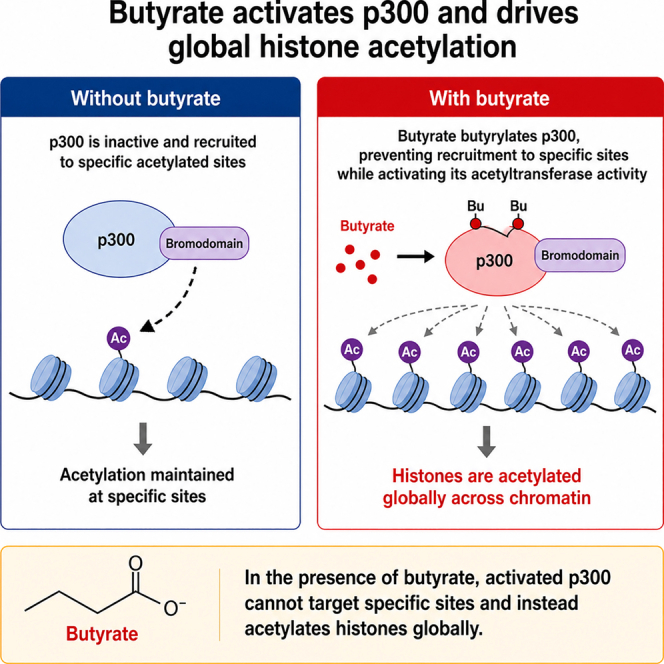

## Teaser

biological sciences; molecular genetics; molecular biology

## Introduction

Short-chain fatty acids (SCFAs) are defined by the presence of an aliphatic tail of one to six carbon atoms, mainly produced from the intestinal microbial fermentation of dietary fiber in the gut. Acetate (C2), propionate (C3), and butyrate (C4) are the main SCFAs, which are produced in the human proximal colon in 70–140 mM concentration.^1^ Butyrate exerts a variety of biological beneficial effects, including enhancing the intestinal epithelial barrier, maintaining intestinal mucosal immunity, anti-inflammation, and reducing inflammatory bowel disease (IBD)-associated symptoms.^2^^,^^3^ Although butyrate also signals through G-protein coupled receptors (GPCRs)^4^^,^^5^ and peroxisome proliferator-activated receptor gamma (PPAR-γ)^6^^,^^7^ and can affect cellular metabolism and intracellular pH,^8^ the current scientific consensus is that butyrate exerts its physiological effects mainly by altering gene expression through epigenetic modifications related to increased histone acetylation. The acetylation of histones is a highly dynamic process regulated by two antagonistic families of enzymes: histone acetyltransferases (HATs) use the substrate acetyl-CoA to covalently link the acetyl group to lysine residues, whereas histone deacetylases (HDACs) stabilize the chromatin structure by removing the acetyl group and restoring the side chain’s positive charge. Therefore, the concerted action of HATs and HDACs collectively orchestrates the dynamic regulation of gene expression through histone modification.

Historically, the increase in histone acetylation by butyrate has largely been attributed to its inhibitory effects on HDACs,^9^ and many studies have shown that butyrate increases histone acetylation in different cell types, presumably via HDAC inhibition.^10^^,^^11^^,^^12^^,^^13^ For example, in human peripheral blood monocyte-derived macrophages, butyrate enhances antimicrobial activity supposedly through specific inhibition of HDAC3, as this phenotype can be copied by siRNA silencing and small molecule inhibitors of HDAC3.^12^

More recently, butyrate was found to also result in the hyperactivation of the HAT p300.^14^ The catalytically activate HAT domain of p300 contains a lysine-rich autoinhibitory loop (AIL) that prevents binding to the histone substrate.^15^ This AIL can become acetylated by another p300 molecule, resulting in the release of the AIL from the substrate-binding pocket and the activation of p300.^15^ Mass spectrometry showed that butyrate can also bind to the AIL, a process called butyrylation, also releasing it and resulting in hyperactivation.^14^

Butyrate-mediated HDAC inhibition and HAT activation have been proposed to result in the acetylation of histones that regulate specific genes in macrophages,^12^ which are key immune cells of the intestinal tract.^16^ However, the precise histone acetylation sites that are affected by butyrate have not yet been mapped in macrophages. In this study, we, therefore, used chromatin immunoprecipitation-sequencing (ChIP-seq) to test the effect of butyrate on histone acetylation in human monocyte-derived macrophages. Surprisingly, this revealed a reduction in specific histone acetylation sites, despite microscopy and western blot showing a clear increase in global histone acetylation. Maintenance of specific histone acetylation sites is thought to involve a positive feedback loop, where p300 is recruited to acetylated histones by its bromodomain to acetylate other sites nearby.^15^ However, it was recently shown by ChIP-seq that only inactive p300 is recruited to acetylated histones.^17^ Indeed, using a combination of molecular dynamics (MD) and immunoprecipitation experiments, we show that AIL acetylation and butyrylation induce a conformational shift in p300, preventing its bromodomain from binding to acetylated histones and abrogating the feedback loop required for the maintenance of specific acetylation sites. Thus, our data show that butyrate blocks specific histone acetylation by preventing recruitment of p300 to acetylated histones.

## Results

### Butyrate decreases specific histone acetylation at transcription start sites

**Figure 1:**
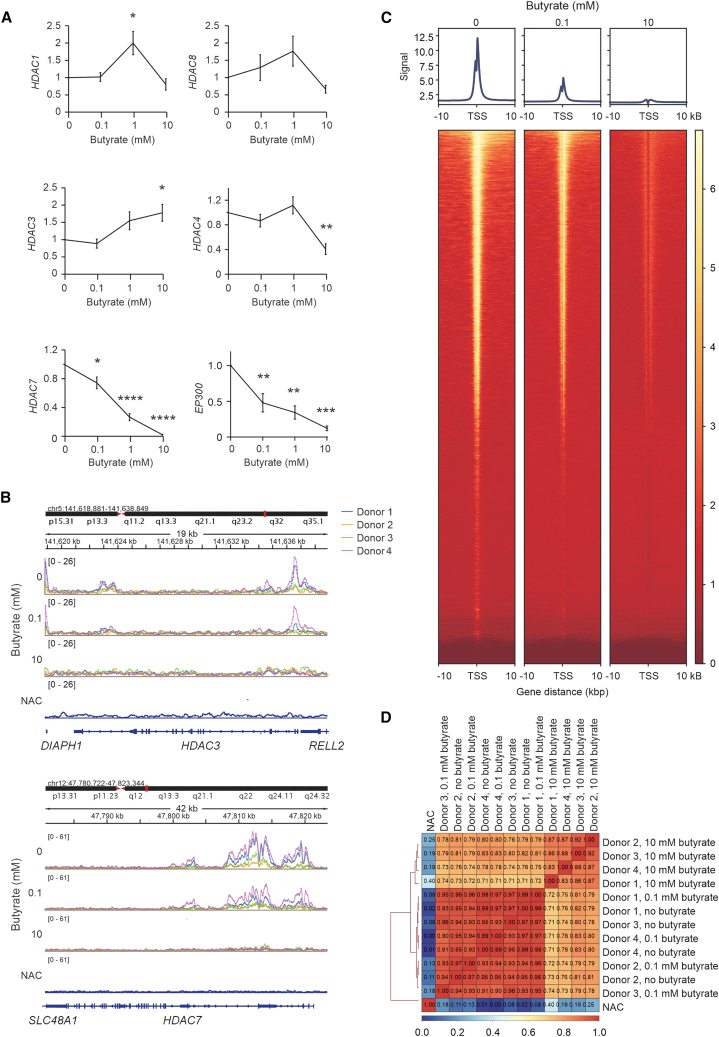
Butyrate decreases the enrichment of specific histone acetylation relative to total histone acetylation (A) *HDAC* mRNA levels by RT-qPCR normalized to without butyrate. One-way ANOVA with Dunnett’s multiple comparisons test, *n* = 3 donors; error bars represent means ± SEM; ∗*p* < 0.05, ∗∗*p* < 0.01, and ∗∗∗∗*p* < 0.0001; conditions were compared with the no butyrate condition. (B) Genome browser snapshots of published ChIP-seq data^21^ showing H3K27Ac enrichment in *HDAC3*, *HDAC7*, and flanking genes. NAC, no antibody control. (C) Intensity profile overlap of all transcription start sites (TSSs), showing a dose-dependent decrease in fold-enrichment of peaks with Na-butyrate. (D) Correlation heatmap of the consensus peaks of the H3K27Ac signal in a window of −1 to 1 kbp around the 1,000 TSS, showing that the 10 mM butyrate condition clusters together (*n* = 4 donors). Human peripheral blood mononuclear-derived macrophages were stimulated for 24 h with LPS, IFN-γ and the indicated concentrations of Na-butyrate.

Since butyrate results in a pronounced transcriptional reprogramming,^12^^,^^18^ we hypothesized that it might not only directly inhibit HDACs or activate HATs but also affect their activity by altering their expression levels. We, therefore, determined the expression levels of the genes coding for HDACs and p300 in human monocyte-derived macrophages that were stimulated for 24 h with the pathogenic stimulus lipopolysaccharide (LPS), the inflammatory cytokine interferon (IFN)-γ, and a broad concentration range of butyrate (0.1–10 mM) using RT-qPCR and RNA-seq. We selected this range as it is reflective of concentrations in different regions of the large intestine^1^ and the concentrations used in other *in vitro* studies.^8^^,^^10^^,^^19^^,^^20^^,^^21^^,^^22^ We normalized gene expression to housekeeping genes *SNRPD3* and *GAPDH* that did not change upon butyrate treatment (Figure S1A). mRNA levels of *HDAC3* were elevated upon increasing concentrations of butyrate, whereas *HDAC7* and *SIRT1* were downregulated and *HDAC1*, *HDAC4*, *HDAC8*, *SIRT2*, and *SIRT3* did not show a clear dose response (Figures 1A, S1B, and S1C). Moreover, we also observed a dose-dependent reduction of *EP300* (the gene coding for p300) and increase of *HAT1* mRNA levels by butyrate (Figures 1A and S1C). Thus, butyrate differentially affects the mRNA levels of these HATs and HDACs.

In addition to its effects on gene expression by epigenetic regulation, HDAC inhibitors such as trichostatin A (TSA) have been reported to post-transcriptionally regulate mRNA levels by decreasing mRNA stability.^23^^,^^24^ We, therefore, assessed whether the differential HDAC expression upon butyrate treatment correlated with histone acetylation at transcription start sites (TSSs) of HDAC-encoding genes by a detailed analysis of our recent ChIP-seq data.^21^ We performed ChIP-seq on histone 3 K27-acetylation (H3K27Ac), as this is a canonical active enhancer mark associated with higher transcriptional activation,^15^ and butyrate is well known to increase H3K27Ac.^12^ For these experiments, we used a polyclonal rabbit antibody raised against H3AcK27 (Diagenode, C15410196), because it has been used extensively in the literature for ChIP-seq. However, more recently, it has been shown to also bind to other lysine acetylation sites of histones, which suggests that our findings could also apply to other histone acetylation sites.

Surprisingly, we observed that butyrate reduced the enrichment of H3K27Ac at the genes coding for all HDACs (relative to total reads), even for *HDAC3*, despite it being higher expressed in the presence of butyrate (Figures 1B and S1D). This was also observed for the genes coding for calprotectin (*S100A8* and *S100A9*) that have been reported to be strongly upregulated by butyrate in this cell type^12^ (Figure S1E). In fact, this was a global phenomenon, as we consistently observed that butyrate dose-dependently reduced the enrichment of H3K27Ac at all TSSs (i.e., throughout the entire genome) for all four donors (Figures 1C, 1D, and S2A). Butyrate also consistently reduced the intensity of common acetylation sites that were present in all four tested donors, identified by peak calling of H3K27ac for the conditions without butyrate (Figure S2B). This reduction in histone acetylation by butyrate was also apparent when we identified regions with significant changes in acetylation levels using a sliding window of 1,000 bp and DESeq2 (Figure S2C). Finally, histone acetylation was also consistently reduced by butyrate on enhancer regions (Figure S2D). Because there is no data available on the locations of enhancer regions in monocyte-derived macrophages, we identified these as H3K4me1 positive, H3K27Ac positive, and H3K4me3 negative regions in their precursors, CD14-positive monocytes, using Encyclopedia of DNA Elements (ENCODE) data. Thus, contrary to our expectations, butyrate dose-dependently decreased H3K27 acetylation at specific sites relative to the entire genome.

### Butyrate increases histone acetylation globally

The ChIP-seq results showed that butyrate reduced the fold-enrichment of H3K27 acetylation peaks at specific sites relative to the entire genome. This seems contrary to the current model that butyrate increases histone acetylation.^9^ However, this conclusion is largely based on bulk experiments that assess histone acetylation of the entire chromatin, mainly by incorporating radioactive acetate, altered electrophoretic mobility of histones, and western blot analysis against acetylated histones.^10^^,^^19^^,^^22^ Therefore, the decrease of histone acetylation at specific sites that we observed by ChIP-seq might be caused by an increase in histone acetylation everywhere else, thus, non-specifically throughout the entire chromatin, as recently shown by microscopy.^25^

**Figure 2:**
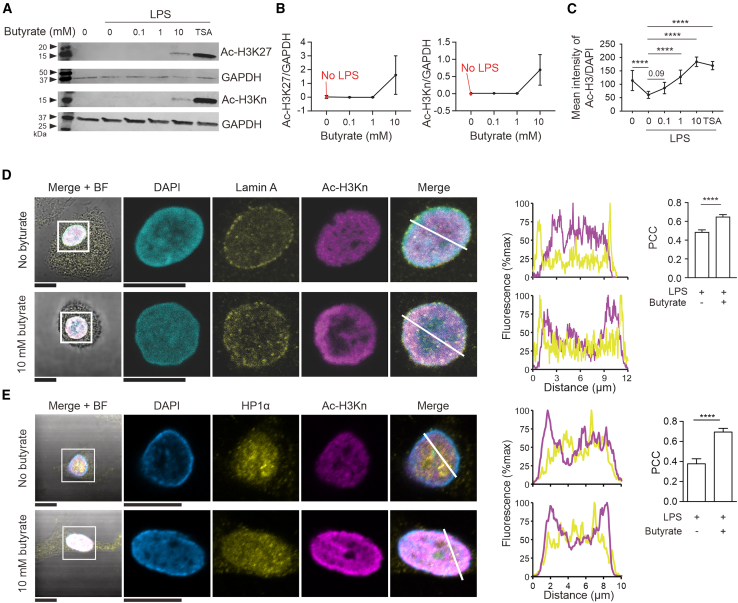
Butyrate induces hyperacetylation of histones globally (A) Western blots showing histone acetylation detected with two different antibodies recognizing acetylated (Ac-)H3K27 and H3K4+9 + 14+18 + 23+27 (Ac-H3Kn) acetylation. (B) Quantification of Ac-H3K27 and Ac-H3Kn levels normalized to GAPDH. *n* = 3 donors; error bars represent mean ± SEM; the complete blots for all donors are shown in Figure S3A. (C‑E) Immunofluorescence microscopy of macrophages labeled for Ac-H3Kn. (C) Quantification of the mean intensity of histone acetylation normalized to DAPI. One-way ANOVA with Tukey’s multiple comparisons test, *n* = 3 donors, at least 5 cells per donor; error bars represent mean ± SEM. ∗∗∗∗*p* < 0.0001; representative confocal images are shown in Figure S3B. The macro for automated image analysis is provided in the supplemental information. (D and E) Representative confocal images showing immunolabeled nuclei for Ac-H3Kn (magenta), DAPI (blue), and Lamin A (yellow, D) or heterochromatin protein 1α (HP1α, yellow, E). The line graphs show fluorescence intensity profiles indicated by the white lines. The bar charts show the Pearson correlation coefficient (PCC) between the H3Ac and Lamin A or HP1α stainings (paired 2-sided *t* tests, *n* = 3 donors, at least 5 cells per donor; error bars represent mean ± SEM. Scale bars, 5 μm). Human peripheral blood mononuclear-derived macrophages were stimulated for 24 h with LPS, IFN-γ, and the indicated concentrations of Na-butyrate or 10 μM trichostatin A (TSA).

To confirm that butyrate increases the acetylation of total histones, we probed lysates of LPS and butyrate-treated macrophages by western blot with the same antibody for H3K27 acetylation as used in our ChIP-seq (Figures 2A, 2B, and S3A). We also probed the blot with an antibody recognizing multiple acetylation sites of H3 (K4, K9, K14, K18, K23, and K27) (Figures 2A, 2B, and S3A). While 0.1 and 1 mM butyrate did not result in detectable levels of acetylated H3 by western blot, 10 mM butyrate showed a clear increase in H3 acetylation, which is consistent with literature.^8^^,^^12^ Moreover, 10 mM butyrate also increased acetylation of H4 (K5, K8, K12, and K16) (Figure 4D), indicating that butyrate increases acetylation of multiple histones. We also attempted to probe for total H3 (Figure S3A). However, we encountered technical difficulties with the H3 antibody, which produced inconsistent staining. This appears to be due in part to incomplete stripping of the Ac-H3K9 and Ac-H3K27 antibodies. Prolonged stripping resulted in overstripping, whereas shorter stripping times led to substantial residual background.

To further address the discrepancy between the ChIP-seq and western blot, we also quantified the levels of acetylated H3 by immunofluorescence microscopy. We observed a clear and dose-dependent increase of H3 acetylation in the nucleus (Figures 2C, 2D, and S3B). While no bands for acetylated H3 were detectable for the 1 mM butyrate condition by western blot, quantification of the immunofluorescence labeling showed a clear increase in H3 acetylation at 1 mM butyrate condition (Figure 2C), likely reflecting a lower detection sensitivity of our western blot assay. In accordance with previous findings,^25^ we noted that the distribution of acetylated histones differed markedly: while in the absence of butyrate, acetylated H3 mainly located in foci that were distributed relatively uniformly throughout the nucleus, it located more toward the edge of the nucleus in the presence of 10 mM butyrate (Figure 2D). As shown previously,^25^ this indicates that butyrate increases acetylation of heterochromatin, since heterochromatin is typically located at the edge of the nucleus and contains a higher density of histones than euchromatin. To quantify this phenotype, we co-stained for acetylated H3 and Lamin A, which is a major component of the nuclear lamina. Indeed, colocalization between these stainings was markedly increased in cells treated with 10 mM butyrate (Figure 2D). Moreover, butyrate also increased the overlap of acetylated H3 with the heterochromatin marker protein heterochromatin protein 1α (HP1α) (Figure 2E).

Finally, we checked for evidence that non-specific H3 acetylation was increased by butyrate in our ChIP-seq experiments. For this, we randomly selected 1,000,000 genomic regions located at least 5 kb away from any annotated gene and calculated the normalized signal within each region (Figure S3C). As expected, we observed the anticipated trend. However, the magnitude of the increase is small, as the signal is distributed across the entire genome, and inter-donor variability is relatively high. Consequently, this effect did not reach statistical significance.

Together, we conclude that butyrate increases the acetylation of total histones throughout the entire chromatin, and thereby, the specific acetylation of histones near TSS and other specific sites is reduced relative to the rest of the chromatin.

### Activation prevents binding of p300’s bromodomain to acetylated histones

Next, we explored the mechanism of how butyrate results in loss of acetylation of specific histones, but increases overall histone acetylation. We focused on p300 for three reasons. First, butyrate has been shown to hyperactivate p300^14^ and the global increase in hyperacetylation is suggestive of a role for hyperactivated p300 instead of selective inhibition of specific HDACs. Second, H3K27 and other lysines on H3, H4, and other histones are main sites for p300 acetylation,^26^ and butyrate-induced hyperacetylation of H3K27 has been shown to be blocked by the p300 inhibitor A485.^14^ Third, maintenance of specific histone acetylation sites is believed to require a positive feedback loop, where p300 is recruited to acetylated histones via its bromodomain, and this, in turn, promotes the acetylation of other histone acetylation sites nearby.^15^ However, it was recently shown by ChIP-seq that only inactive p300 is recruited to acetylated histones,^17^ suggesting that the butyrate-induced hyperactivation of p300 might disable this positive feedback loop and thus, prevent the maintenance of specific histone acetylation sites.

Indeed, comparison of a cryo-EM structure of the p300 catalytic core bound to the acetylated H4 nucleosome^27^ with a cryo-EM structure of free p300^28^ suggests that the inhibitory RING-loop, located in p300’s regulatory plant homeodomain (PHD) that connects the bromodomain with the HAT domain, undergoes a conformational change and moves between the HAT and the bromodomains upon activation (Figure S4). Moreover, it was shown by mass spectrometry that lysine residues located not only in the AIL but also in this RING-loop are autoacetylated.^29^ This acetylation might affect the interactions of the RING-loop with the HAT and bromodomains. We, therefore, hypothesized that acetylation or butyrylation of the AIL would promote the interaction of the RING-loop with the bromodomain, thereby preventing its binding to acetylated histone substrates.

**Figure 3:**
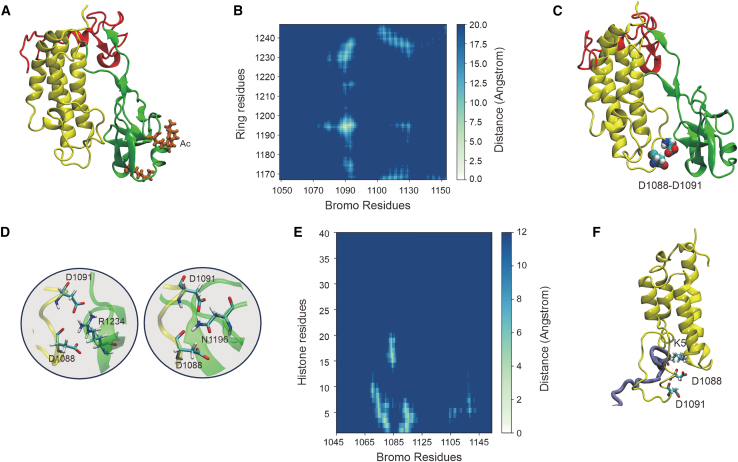
Molecular dynamics simulations indicate that p300’s RING-loop can block binding of p300’s bromodomain to acetylated histones (A) Structure extracted from the simulation of the triple mutant K1180Ac, K1203Ac, and K1228Ac, with acetylated lysines highlighted in orange. The bromodomain and RING-loop are in contact. (B) Contact map between the RING-loop and bromodomain residues, averaged over the last 500 ns of simulation. (C) Structure detailing the interaction between the bromodomain and RING-loop, showing that residues D1088 and D1091 are primarily involved in binding. (D) Interaction between residues D1088 and D1091 (bromodomain) with N1196 and R1234 (RING-loop). (E and F) MD simulations of p300 with the bromodomain bound to the N-terminal chain of histone H4 (ice blue). The reference structure is PDB 8HAG. The contact map between the bromodomain and histone protein chain residues is shown, along with a representative structure extracted from the simulation. The reference structure is PDB 6GYR (A–D). Different protein sub-units are color-coded: bromodomain (yellow), RING-loop (green), and PHD domain (red).

We first conducted MD simulations of p300 to test this hypothesis. The MD simulations were based on a reference structure (PDB: 6GYR)^30^ of p300 containing the bromodomain, PHD-domain (including RING-loop), and HAT (residues 1,045–1,664). As we were specifically interested in the interactions between the bromodomain and RING-loop upon acetylation, we performed the first set of simulations on a smaller system composed of the bromodomain and PHD-domain (Figures 3A, 3B, and S5). Besides the non-acetylated form, we simulated the triple acetylated form K1180Ac, K1203Ac, and K1228Ac, with three lysines in the RING-loop that have been shown to be acetylated.^29^

Based on a 1 μs simulation, we generated a contact map between the bromodomain and RING-loop. The bromodomain interacted stably with the RING-loop when the lysines were acetylated, but this interaction did not take place in the non-acetylated system (Figure S5). However, this interaction is not directly mediated by the acetylated lysines. Instead, simulations of the complete non-acetylated system (Figure S6) revealed that the charged lysines and aspartates participate in a network of interactions between the RING-loop and HAT. When these lysines are neutralized by acetylation, these interactions are disrupted, allowing the RING-loop to detach from the HAT and move closer to the bromodomain.

Focusing on the interactions between the bromodomain and RING-loop, we found that this involves a specific segment of the bromodomain (loop 1,080–1,095). Notably, two aspartate residues (D1088 and D1091) in the bromodomain contact arginine and asparagine residues in the RING-loop (N1196 and R1234), as shown in representative structures and contact plots over time from the simulation (Figures 3C, 3D, and S7). These aspartates are located on a highly flexible loop (see root-mean-square fluctuation [RMSF] in Figure S7A), which adapts to form the binding region for the histone peptide chain. The interaction of D1088 and D1091 with the RING-loop could, therefore, compete with the binding of acetylated histones.

In the structure of p300 bound to the nucleosome (PDB: 8HAG),^27^ the ten N-terminal residues of H4 remain unresolved. To clarify whether residues D1088 and D1091 of the bromodomain interact with the histone peptide, we reconstructed the system using the reference structure 8HAG. Here, the peptide chain of H4 containing the ten missing N-terminal residues was rebuilt. Reconstructing the N-terminal is essential for maintaining the stability of the histone-bromodomain complex. Omitting this reconstruction would exclude potential interactions between the N-terminal and the bromodomain, which are likely to be biologically significant. The model reconstruction process is discussed in detail in the STAR Methods. Briefly, the model with the highest score was selected and underwent a comprehensive procedure of energy minimization, equilibration, and microsecond-long MD simulations. The results of this analysis are presented in Figures 3E and 3F. Here, we show a contact map between the bromodomain and the first 40 residues of the histone peptide chain, along with a representative structure illustrating this interaction. The first 15 residues of the peptide are stably bound to the bromodomain, with acetylated lysine 12 buried in the bromodomain. The preceding residues wrap around the bromodomain, including the region spanning residues 1,090–1,100. This revealed the interaction between K5 and D1091 (Figure S8).

The inherent limitations of the prediction are acknowledged, particularly due to the flexibility and dynamic nature of the N-terminal region. The accuracy of this inferred region is heavily influenced by the structural context provided by the available template and may not fully capture the conformational heterogeneity that could exist in a physiological environment. However, confidence in the reliability of the generated models is supported by the availability of the full protein structure, with the N-terminal histone region already in contact with the bromodomain via residue G11 (see Figure S8A). The model is further validated by favorable scoring results and the interactions detected, which are the outcome of extensive MD sampling, confirming the reliability of the predicted structure.

In conclusion, the MD simulations indicate that the bromodomain interacts with either the RING-loop or the histone peptide chain using the same residues, suggesting that binding to one excludes binding to the other. These results, therefore, suggest that if the bromodomain and RING-loop contact through aspartates D1088 and D1091 of the bromodomain, this prevents binding with the histone chain. Alternatively, as the RING-loop approaches the bromodomain, the resulting interactions could weaken the binding with the histone peptide, causing it to detach. As explained earlier, the binding of the bromodomain to acetylated histones is believed to allow for the maintenance of specific histone acetylation sites by positive feedback.^15^ As butyrate results in the butyrylation of p300’s AIL,^14^ the loss of specific histone acetylation peaks in butyrate-treated cells might be caused by the loss of p300 binding to acetylated histones.

### Butyrylated p300 hyperacetylates histones globally

**Figure 4:**
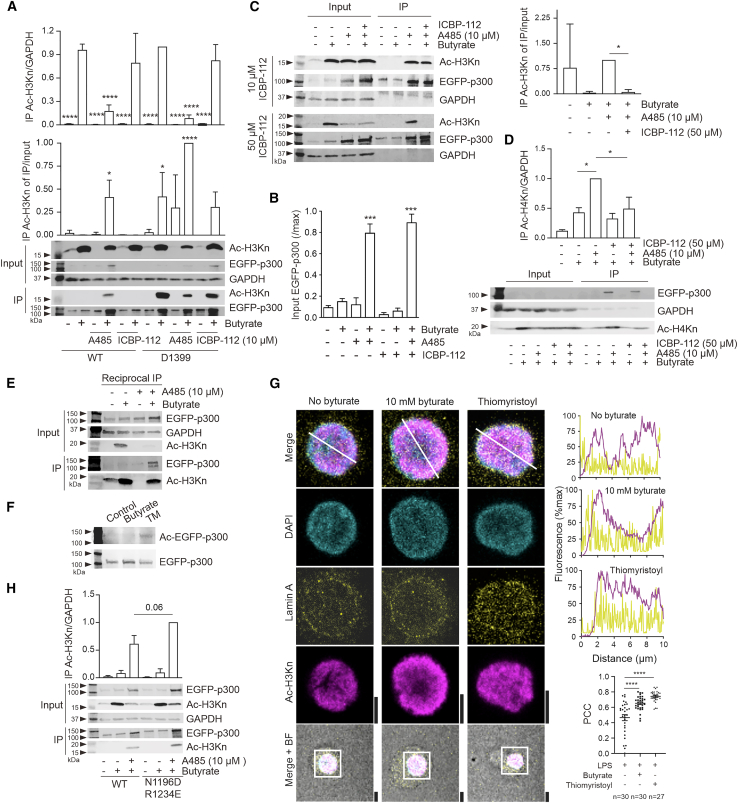
Butyrylated p300 does not bind to acetylated H3 (A) HeLa cells were transfected with the EGFP-tagged catalytic core of p300 wild type (WT) or catalytically inactive D1399Y mutant and stimulated for 24 h with 10 mM Na-butyrate, 10 μM HAT inhibitor A485, and/or 10 μM bromodomain inhibitor ICBP-112 prior to immunoprecipitation (IP) for EGFP. Western blot showing Ac-H3K4+9 + 14+18 + 23+27 (Ac-H3Kn), EGFP-p300, and GAPDH detected in lysates and IP samples. Top graph: quantification of Ac-H3Kn levels normalized to GAPDH in lysates. Bottom graph: quantification of Ac-H3Kn levels in IP samples normalized to lysates. One-way ANOVA with Dunnett’s multiple comparisons test; *n* = 3 independent experiments; error bars represent mean ± SEM; ∗*p* < 0.05 and ∗∗∗∗*p* < 0.0001; each condition was compared with the butyrate-treated WT condition. (B) Quantification of EGFP-p300 in the input samples of the IP experiments in (A), (C), (D), and (H). Repeated measures ANOVA; each condition was compared with the butyrate-treated WT condition. (C) Transfected HeLa cells were stimulated for 24 h with 10 mM Na-butyrate, 10 μM A485, and 10 or 50 μM ICBP-112 prior to IP for EGFP, showing that A485-induced binding of p300 to acetylated histones could be blocked by 50 μM ICBP-112. Graph: quantification of Ac-H3Kn levels in IP samples normalized to lysates. One-way ANOVA with Tukey’s multiple comparisons test; *n* = 3 independent experiments, error bars represent mean ± SEM. ∗*p* < 0.05. (D) Transfected HeLa cells were stimulated for 24 h with 10 mM Na-butyrate, 10 μM A485, and 50 μM ICBP-112. Western blot showing Ac-H4K5+8 + 12+16 (Ac-H4Kn) in lysates and IP samples with antibody against EGFP. Graph: quantification of Ac-H4Kn levels in IP samples normalized to GAPDH in lysates. One-way ANOVA with Tukey’s multiple comparisons test; *n* = 3 independent experiments, error bars represent mean ± SEM. ∗*p* < 0.05. (E) Reciprocal IP as in (A), using IP with antibody against Ac-H3Kn. (F) Transfected HeLa cells were stimulated for 24 h with 10 mM Na-butyrate and 10 μM of SIRT2 inhibitor thiomyristoyl (TM). Western blot probed with an antibody recognizing an acetylated lysine located in the AIL of p300 (K1542), showing that acetylated p300 was increased by TM treatment. (G) Human peripheral blood mononuclear-derived macrophages were stimulated for 24 h with LPS, IFN-γ, and 10 mM Na-butyrate or 10 μM TM. Representative confocal images showing immunolabeled nuclei for Ac-H3Kn (magenta), Lamin A (yellow), and DAPI (cyan). Scale bars, 5 μm. The line graphs show fluorescence intensity profiles indicated by the white line. The graph shows the Pearson correlation coefficient (PCC) between the Lamin A and Ac-H3Kn staining. One-way ANOVA with Dunnett’s multiple comparisons test; 3 donors were analyzed, >8 cells per donor (exact counts in figure); error bars represent mean ± SEM; ∗∗∗∗*p* < 0.0001. More representative cells are shown in Figure S9G). The macro for automated image analysis is provided in the supplemental information. (H) HeLa cells were transfected with N1196D and R1234E mutant and stimulated for 24 h with 10 mM Na-butyrate and 10 μM HAT inhibitor A485, followed by IP for EGFP. Quantification of Ac-H3Kn levels normalized to GAPDH in lysates. One-way ANOVA with Tukey’s multiple comparisons test; *n* = 3 independent experiments, error bars represent mean ± SEM. Complete blots for all experiments are shown in Figures S9 and S10.

We tested the prediction from the MD simulations that acetylation or butyrylation of p300’s AIL reduces the binding of its bromodomain to acetylated histones. We tested this by assessing the effect of butyrate on the binding of p300 to acetylated H3 through immunoprecipitation experiments. As the levels of endogenous p300 in human monocyte-derived macrophages were too low for immunoprecipitation experiments, and these cells are difficult to transfect with high efficiency, we overexpressed GFP-tagged residues 1,048–1,664 of p300, consisting of its bromodomain, PHD with RING-loop, and HAT domain, in HeLa cells.^31^ Western blot on complete cell lysates (inputs) showed that 10 mM butyrate increased H3 and H4 acetylation in the HeLa cells, which could be partially inhibited by the HAT inhibitor A485 that binds to the catalytic site of the HAT domain^32^ but not by 10 μM of the bromodomain inhibitor ICBP-112 (Figures 4A–4C, S9A, and S9B). A485 treatment did not completely block butyrate-induced histone acetylation, likely because of the activity of other HATs and butyrate’s inhibition of HDACs.

In line with the predictions from the MD simulations and previous ChIP-seq data,^17^ A485 treatment resulted in stable binding of p300 to acetylated H3 and H4 histones, as shown by both immunoprecipitation with an antibody against GFP and the reciprocal immunoprecipitation with an antibody against acetylated H3 (Figures 4A–4E and S9A–S9E). A485 treatment also increased cellular levels of the EGFP-p300 construct (Figure 4B), potentially because histone association protects p300 from cytoplasmic degradation. Unfortunately, we could not consistently detect endogenous p300 in our western blot, likely due to unreliable transfer caused by its very large molecular weight (364 kDa) and/or low abundance.

A catalytically inactive mutant of p300, carrying the canonical D1399Y mutation located within the catalytical center of the HAT domain,^15^ also bound stably to acetylated H3 (Figures 4A and S9A). While A485-induced histone binding by p300 could not be blocked by 10 μM ICBP-112, this binding was reduced by 50 μM ICBP-112 (Figure 4C; Figures S9B and S9C). Similar results were observed for acetylated H4 (Figures 4E and S9D). Thus, only catalytically inactive p300 stably binds to acetylated histones via its bromodomain, whereas activated p300 (i.e., with acetylated or butyrylated AIL) does not.

To confirm that the hyperactivation of p300 results in the loss of specific histone acetylation, we performed experiments with thiomyristoyl, a small molecule inhibitor of sirtuin 2 (SIRT2). SIRT2 is a class III HDAC that deactivates p300 by deacetylating its AIL.^29^ While other HDACs can also deacetylate p300, SIRT2 plays a key role in this process, as its inhibition by thiomyristoyl has been shown to increase AIL acetylation.^29^ This was confirmed by western blot using an antibody specific for acetylated K1542 in the AIL of p300, which demonstrated that thiomyristoyl treatment enhanced AIL acetylation of overexpressed p300 in HeLa cells (Figures 4F and S9F). Moreover, immunofluorescence microscopy of human monocyte-derived macrophages showed that acetylated H3 located more strongly toward the nuclear rim upon thiomyristoyl treatment, thus phenocopying the effect of butyrate treatment (Figures 4G and S9G). These findings strengthen our conclusion that p300 with acetylated or butyrylated AIL is not recruited to acetylated histones.

To test the prediction from the MD simulation that the interaction of D1088 and D1091 with the RING-loop competes with the binding of acetylated histones, we mutated the N1196 and R1234 residues of the RING-loop and performed immunoprecipitation experiments on HeLa cells. Conversion of only the N1196 residue into aspartic acid had no effect. However, as predicted by our MD simulations, western blot results showed that upon co-treatment with butyrate and A485, the p300 double mutant N1196D and R1234E showed a trend to binding stronger to acetylated histones compared with wild type (Figures 4H and S10). However, this effect was small (∼35%) and did not reach significance (*p* = 0.06), potentially because the mutation did not completely disrupt the interaction of the RING-loop with the bromodomain. Therefore, we also tested the D1088R and D1091R double mutant of p300, as we expected these mutations to enhance binding to acetylated histones even further. However, this mutant did not express, potentially because of cellular toxicity or misfolding. Similarly, we achieved very low expression of p300 carrying mutations K1180R, K1203R, and K1228R with disrupted acetylation, which were predicted to result in stable binding of the bromodomain to acetylated histones, suggestive of cytotoxicity.

Together, our findings show that butyrate results in loss of acetylation of specific histones, but increases overall histone acetylation, which is in line with previous findings.^25^ Mechanistically, our MD simulations and immunoprecipitation experiments show that only inactive p300 stably binds to acetylated histones through its bromodomain, in accordance with previous ChIP-seq experiments that suggest that inhibition of p300 increases its binding to histone acetylation sites.^17^ The activation of p300 by acetylation or butyrylation results in dissociation of the bromodomain from acetylated histones. Since the recruitment of p300 to acetylated histones is believed to allow for the maintenance of specific histone acetylation sites by positive feedback,^15^ the loss of specific histone acetylation peaks in butyrate-treated cells is likely caused by the loss of p300 binding to acetylated histones.

## Discussion

Butyrate is well known to exert beneficial biological functions by promoting histone acetylation at promoter and enhancer regions of specific genes.^2^^,^^3^^,^^9^ However, our data now show that this model is incomplete: butyrate-mediated histone acetylation is not site-specific but instead, dose-dependently increases histone acetylation globally over the entire chromatin.

**Figure 5:**
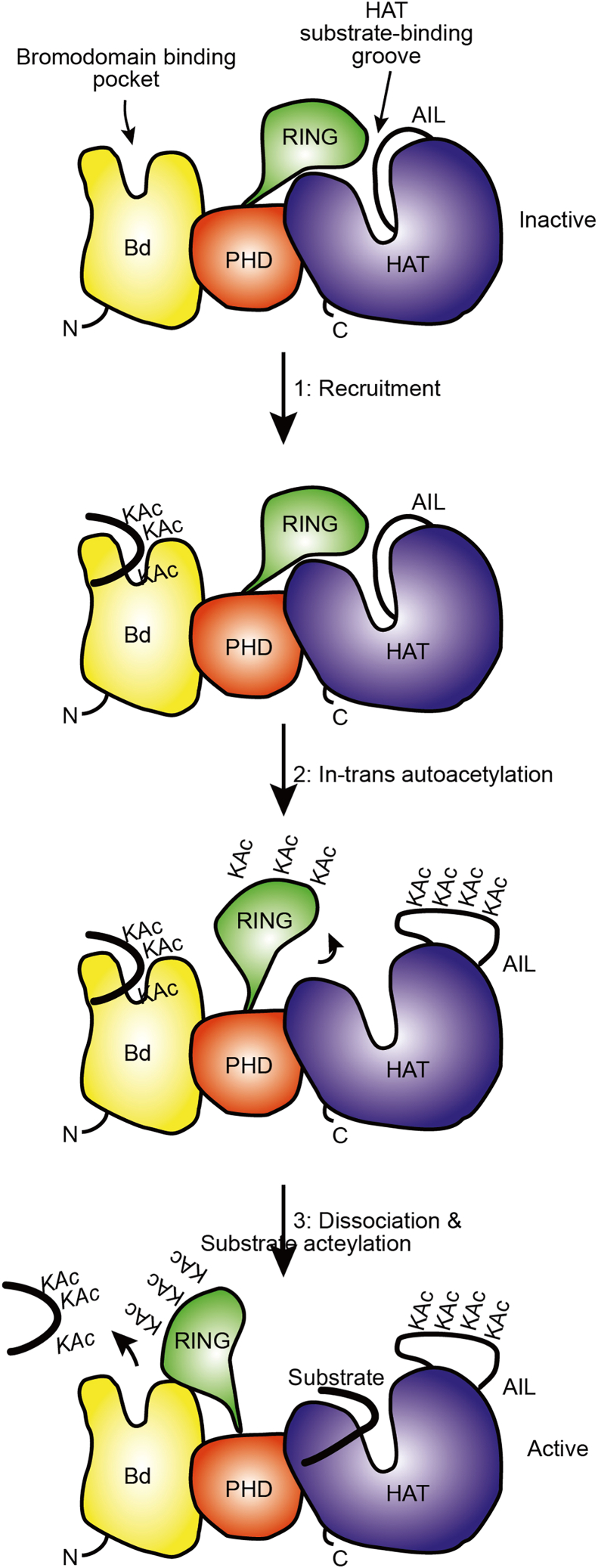
Model for p300 regulation and substrate acetylation In the inactive state (top), the RING-loops blocks the catalytic site of the HAT domain.^33^ The bromodomain of p300 is recruited to acetylated histones (step 1). As proposed previously, the recruitment of at least two copies of p300 result in the in-trans autoacetylation of the autoinhibitory loop (AIL) and displacement of the AIL and RING-loop, thereby exposing the catalytic site of the HAT domain and activating substrate acetylation (step 2).^15^ Lysine residues on the RING-loop also become autoacetylated,^29^ which weakens interactions between the RING-loop and the HAT. The RING-loop now interacts with the bromodomain, causing its dissociation from acetylated histones (step 3). Yellow, bromodomain (Bd); green, RING-loop; red, PHD-domain; blue, HAT; KAc, acetyl-lysine. Model adapted from Delvecchio et al.^15^

Mechanistically, we show that only inactive p300 without acetylated AIL is recruited to acetylated histones via its bromodomain. As the binding of multiple p300 molecules to chromatin is well known to result in p300 activation by trans-acetylation of its AIL,^30^ findings by us and others^17^ suggest that inactive p300 is recruited to acetylated histones and subsequently dissociates upon its activation (Figure 5). This dissociation of active p300 likely allows for the acetylation of other histones nearby, and thereby for maintenance of acetylated histone sites through positive feedback.^15^ However, this feedback cannot occur in the presence of butyrate, since butyrate activates p300 by butyrylation of its AIL,^14^ and p300 is, therefore, no longer recruited to acetylated histones. As a consequence, butyrylated p300 acetylates histones at random, resulting in the global hyperacetylation observed by us and others.^25^ This effect of butyrate can be phenocopied by the inhibition of SIRT2, which prevents deactivation of p300. Moreover, since *in vitro* activity assays showed that SIRT2 is about 4-fold less active toward butyrylated than acetylated substrates,^34^ SIRT2 will likely less efficiently deactivate butyrylated p300, which could further increase the global histone hyperacetylation. A scheme of the proposed mechanism is shown in Figure S11.

Since hyperactivated p300 no longer selectively binds acetylated histones, it is reasonable to expect that it will also increase acetylation of non-histone substrates. p300 is known to acetylate a broad range of non-histone proteins, including many key transcription factors and signaling molecules.^35^ However, whether butyrate induces aspecific acetylation of non-histone substrates remains an untested hypothesis that will need to be addressed in future studies. In addition, it will be interesting to confirm the effects of butyrate on p300 acetyltransferase activity *ex cellulo*.

Our study contrasts many studies where, through ChIP-qPCR and ChIP-seq analyses, it is found that butyrate increases histone acetylation at specific promoter sites.^36^ These differences are unlikely to be the result of different butyrate concentrations, as we already observed the increase in global histone acetylation at 0.1 mM butyrate by microscopy, which is lower than that used in most studies, and the effect is highly pronounced at 10 mM, which is higher than that used in most studies. Notably, this increase in histone acetylation was not detected by western blot, likely due to insufficient sensitivity. Although we cannot rule out cell-type-specific effects, we believe our mechanism is general, given the key role of p300 (and its close paralog CBP) in most cell types. Moreover, ChIP-qPCR might be prone to two experimental artifacts. First, ChIP-qPCR data are usually normalized against the input, but as we show that butyrate increases the acetylation of histones globally, this might result in higher levels of immunoprecipitated genes, especially with low DNA fragmentation. Second, ChIP data are sometimes normalized against other genes that are not expected to be altered upon butyrate treatment, such as *RPL13*, *CKS2*, and *GAPDH*. However, our ChIP-seq data show that butyrate also affects histone acetylation at those genes (Figure S1F). In line with this, our ChIP-qPCR data showed a reduction in histone acetylation of *CKS2* and *GAPDH* by 0.1 and 10 mM butyrate (Figure S1G).

The 0.1–10 mM butyrate concentrations used in our study are high and are present only in the colon.^1^ Given that the effect on histone modifications is much less pronounced at 0.1 mM butyrate compared with 10 mM, we do not expect to observe such effects at micromolar concentrations. In fact, we have recently shown that butyrate exerts opposite effects on macrophages depending on its concentration. At sub-mM concentrations, it is anti-inflammatory, suppressing the production of pro-inflammatory cytokines while increasing the production of the anti-inflammatory cytokine IL-10; in contrast, at millimolar concentrations, butyrate promotes the production of pro-inflammatory cytokines.^21^ We further demonstrated that the effects observed at low concentrations are mediated by mechanisms distinct from histone modification, notably signaling through GPCRs and CD36. As proposed in our previous manuscript,^21^ these findings suggest that butyrate may function as a signaling molecule involved in intestinal integrity, as disruption of the intestinal barrier would expose macrophages to high local concentrations of butyrate.

A question that is raised by our study is how butyrate can result in both up- and downregulation of specific genes, especially as it has been shown by using pertussis toxin and overexpression that this is independent to GPCR signaling in macrophages.^12^^,^^20^^,^^21^ In addition to its effects on HATs, HDACs, and GPCRs, butyrate has been reported to activate the PPAR-γ, a nuclear receptor that functions as a transcription factor and can regulate the expression of multiple genes.^6^^,^^7^ Moreover, especially at higher concentrations, butyrate promotes fatty acid oxidation and thus alters metabolic signaling cascades.^3^ We recently showed that at acidic extracellular pH, butyrate is also accumulated within cells and can acidify the cells.^8^ The beneficial biological functions exerted by butyrate can likely be attributed to the combination of global histone acetylation, GPCR and PPAR signaling, and metabolic and pH effects. Our data underscore the complexity of butyrate-mediated regulation of metabolic and immune functions, which cannot be attributed solely to epigenetic control of specific genes via histone acetylation.

### Limitations of the study

A limitation of this study is that the findings are primarily based on *in vitro* experiments using human monocyte-derived macrophages and HeLa cells, combined with MD simulations. Although these approaches provide mechanistic insight into how butyrate affects p300 activity and histone acetylation, the physiological relevance of these mechanisms remains to be validated *in vivo*, particularly within the complex butyrate gradients present in intestinal tissues.

In addition, the ChIP-seq analysis measures relative enrichment rather than absolute acetylation levels, which may complicate interpretation under conditions of global histone hyperacetylation. Finally, several mechanistic experiments relied on the overexpression of truncated p300 constructs in HeLa cells, which may not fully reflect endogenous p300 regulation in primary immune cells.

## Resource availability

### Lead contact

Further information and requests for resources and reagents should be directed to and will be fulfilled by the lead contact, Dr. Geert van den Bogaart (g.van.den.bogaart@rug.nl).

### Materials availability

Plasmids generated in this study have been deposited to Addgene (p300_N1196D_R1234E_GFP, plasmid number #258517).

### Data and code availability

• ChIP-seq and RNA-seq data have been deposited at the Gene Expression Omnibus (GEO) genomics data repository with accession numbers GSE255090 and GSE272870. The other primary data have been deposited at Zenodo (https://doi.org/10.5281/zenodo.14814769).
• This paper does not report original code.
• Any additional information required to reanalyze the data reported in this paper are available from the lead contact upon request.

## Acknowledgments

We thank the Center for Information Technology of the University of Groningen for their support and for providing access to the Hábrók high performance computing cluster and Prof. Dr. Daniel Gerlich from Vienna BioCenter of the Institute of Molecular Biotechnology of the Austrian Academy of Sciences for plasmids. This work was supported by the European Research Council under the European Union’s Horizon 2020 research and innovation programme (grant agreement no. 862137) and China Scholarship Council PhD Programme (CSC202006170020).

## Author contributions

M.J. did the macrophage preparation for ChIP-seq, RT-qPCR, immunoprecipitation, western blot, and confocal microscopy; M.G.C. and S.-J.M. performed the MD simulations; R.M. and I.H.J. performed the ChIP-seq; D.I. analyzed the ChIP-seq data; A.P. and J.L. contributed to the immunoprecipitation, mutagenesis, and microscopy experiments; M.J. analyzed data and wrote the original draft with G.v.d.B.; M.J., G.v.d.B., and F.B. conceptualized the study. All authors commented on the final draft.

## Declaration of interests

The authors declare no conflicts of interests.

## STAR★Methods

### Key resources table

**Table:** 

REAGENT or RESOURCE	SOURCE	IDENTIFIER
**Antibodies**		

Rabbit polyclonal anti-H3AcK4+9 + 14+18 + 23+27 (Ac-H3Kn)	Abcam	Cat#: ab300641, RRID: AB_3107191
Rabbit polyclonal anti-H3K27Ac	Diagenode	Cat#: C15410174, RRID: RRID:AB_2716835
Mouse monoclonal anti-H3, clone 1G1	Santa Cruz	Cat#: sc-517576, RRID: AB_2848194
Rabbit polyclonal anti-H4AcK5+8 + 12+16 (Ac-H4Kn)	Abcam	Cat#: ab177790, RRID: AB_2732882
Rabbit polyclonal anti- Acetyl-EP300-Lys1542	St ’John’s Laboratory	Cat#: STJ97696
Mouse monoclonal anti-GAPDH, clone G-9	Santa Cruz	Cat#: sc-365062, RRID: AB_10847862
Donkey anti-Rabbit IgG IRDye680	LI-COR	Cat#: 926–32223, RRID: AB_621845
Donkey anti-Mouse IgG IRDye680	LI-COR	Cat#: 926–32222, RRID: AB_621844
Mouse monoclonal anti-Lamin A/C (BD)	BD Biosciences	Cat#: 612163, RRID:AB_399534
monoclonal mouse GFP (, 11814460001)	Roche	Cat#: 11814460001, RRID: AB_390913
Rabbit polyclonal anti-GFP	Rockland	Cat#: 600-401-215, RRID: AB_828167)
Mouse monoclonal anti-HP1α, clone 3A11F8	Abcam	Cat#: ab234085, RRID: AB_3677674
Donkey anti-Rabbit IgG (H + L) Alexa Fluor 647 ()	Invitrogen	Cat#: A31573, RRID: AB_2536183
Donkey anti-Mouse IgG (H + L) Alexa Fluor 488	Invitrogen	Cat#: A21202, RRID: AB_141607

**Bacterial and virus strains**		

N/A		

**Biological samples**		

Human blood buffy coat	Dutch Blood Bank	Buffy coat NVT

**Chemicals, peptides, and recombinant proteins**		

CD14 MicroBeads	Miltenyi	Cat#: 130-097-052
M-CSF	RnD Systems	Cat#: 216-MC
Na-butyrate	Sigma Aldrich	Cat#: 820236, CAS: 68917-50-0
Trichostatin A (TSA)	Sigma Aldrich	Cat#: T1952, CAS: 58880-19-6
I-CBP112	Sigma Aldrich	Cat#: SML1134, CAS: 1640282-31-0
Thiomyristoyl	Selleck Chemicals	Cat#: S8245, CAS: 1429749-41-6
A485	Selleck Chemicals	Cat#: S8740, CAS: 1889279-16-6

**Critical commercial assays**		

NEBNext Ultra II RNA Library Prep Kit for Illumina	New England Biolabs	Cat#: E7770L

**Deposited data**		

Raw and analyzed ChIP-seq data	This paper	GEO: GSE255090
Raw and analyzed RNA-seq data	This paper	GEO: GSE272870
Raw and analyzed other data	This paper	https://doi.org/10.5281/zenodo.14814769

**Experimental models: Cell lines**		

Human: HeLa cells	ATCC	RRID: CVCL_0030

**Experimental models: Organisms/strains**		

N/A		

**Oligonucleotides**		

See Table S1		

**Recombinant DNA**		

Plasmid: eGFP-p300	Schneider et al.^31^	Addgene Plasmid #191764
Plasmid: eGFP-p300	Schneider et al.^31^	Addgene Plasmid #191763

**Software and algorithms**		

Bowtie	Langmead et al.^37^	https://bio.tools/bowtie
deepTools	Ramírez et al.^38^	https://deeptools.readthedocs.io/en/develop/index.html
DESeq2	Love et al.^39^	https://bioconductor.org/packages/release/bioc/html/DESeq2.html
STAR	Dobin et al.^40^	https://github.com/alexdobin/STAR
Fiji ImageJ	Schindelin et al.^41^	https://imagej.net/software/fiji/
GraphPad Prism	GraphPad Software Inc	https://www.graphpad.com/
GROMACS	Páll et al.^42^	https://www.gromacs.org/index.html

**Other**		

N/A		

### Experimental model and study participant details

HeLa cells (female cell line; RRID: CVCL_0030; authenticated by ATCC through their human STR profiling cell authentication service) were cultured in Dulbecco’s Modified Eagle’s Medium (DMEM, Gibco) supplemented with 10% fetal bovine serum (FBS, Thermo Fisher Scientific), 1% antibiotic-antimitotic solution (AA, Gibco), and 1% L-glutamine (Gibco) at 37°C and 5% CO_2_. No effect of biological sex on the observed outcomes is expected, as p300 expression does not differ substantially between males and females. Cells were tested for mycoplasm.

Peripheral blood mononuclear cells (PBMC) were obtained from buffy coats of human blood donors (random sex and age) by ficoll gradient centrifugation. Buffy coats were obtained anonymized from the Dutch blood bank. All blood donors were informed about the research and granted their consent to the blood bank. The research is exempt from ethical approval by the Dutch Medical Research with Human Subjects Law, because samples were provided fully anonymized to the researchers and the blood donors did not have to undergo extra procedures.

CD14^+^ monocytes were isolated from PBMC using CD14 MicroBeads (Miltenyi, 130-097-052). Human peripheral blood mononuclear-derived macrophages were generated in Ultra-low adherent 6-well plates (Corning) in 2 mL RPMI 1640 medium (Gibco) supplemented 10% FBS, 1% antibiotic-antimitotic solution (AA, Gibco), and 1% L-glutamine (Gibco) and M-CSF (RnD 216-MC, 100 ng/mL) at 37°C and 5% CO_2_ for seven days. On day 4, macrophages were supplemented with 1 mL medium containing 50 ng/mL M-CSF. For the experiments with butyrate, we used Na-butyrate (Sigma, 820236) and verified the pH.

### Method details

#### RT-qPCR

1×10^6^ human peripheral blood mononuclear-derived macrophages per condition were treated with 1 μg/mL LPS, 20 ng/mL IFN-γ and 0.1 mM, 1 mM or 10 mM sodium butyrate (Sigma) for 24 h. Cells were lysed and then RNA was isolated from the lysate using Quick-RNA Miniprep Kit (ZYMO), cDNA was synthesized with random hexamer primers (Roche) and the M-MLV Reverse Transcriptase kit (ThermoFisher). RT-qPCR was performed using 4 ng of cDNA with PowerUP SYBR Green Master Mix, and the 10 μL for each reaction was incubated at 50°C for 2 min, 95°C for 2 min, followed by 40 cycles of denaturation (95°C for 15 s), annealing and extension (60°C for 1 min) with a BioRad CFX96 qPCR System. The primer sequences are listed in Table S1. Analyzed genes were normalized to Small Nuclear Ribonucleoprotein D3 Polypeptide (SNRPD3).

#### ChIP-seq

The ChIP-seq was performed as described.^21^ 1×10^6^ human peripheral blood mononuclear-derived macrophages per condition were treated with 1 μg/mL LPS, 20 ng/mL IFN-γ and 0.1 mM or 10 mM sodium butyrate for 24 h.

For cross-linking and shearing of chromatin we used the commercially available truChIP Chromatin Shearing Kit (Covaris, cat; PN 520154). For 1×10^6^ human peripheral blood monocyte-derived macrophages we established the optimal formaldehyde fixation time to be 5 min and the optimal shearing time to be 10 or 12 min. We used the “low cell protocol” in the truChIP Chromatin Shearing Kit which is optimized for chromatin shearing of 1–3 million cells using the microTUBE-130 with AFA fiber screw cap (Covaris, 520216). The shearing was done by using the Covaris Focused-ultrasonicator (S220). The immunoprecipitation was carried out based on the standard 2014 version Blueprint Histone ChIP and Kapa Hyper Prep kit protocol described in detail below. Antibody concentration used was 1 μg H3K27ac antibody per IP (cat: C15410174), obtained from Diagenode (Liege, Belgium). After sonification we finished with a total volume of 130 μL chromatin. This was split into two fractions of 65 μL. The first fraction was diluted prior to immunoprecipitation to equilibrate the sheared chromatin in a buffer that is compatible with antibody binding. We added 229 μL ChIP dilution buffer (1% Triton X-100, 1.2 mM EDTA, 16.7 mM Tris (pH 8), 167 mM NaCl) including the AB of interest and 6 μl 50x protease inhibitor cocktail (Roche, cat; 11697498001) resulting in a total volume of 300 μL. The chromatin–antibody mixture was incubated overnight at 4°C under constant rotation. The second – 65 μL - fraction was used as negative control and was kept at 4°C for later use. The next day, for each IP sample 10 μL protein A beads (Thermo scientific, cat: 10001D) and 10 μL protein G beads (Thermo scientific, cat: 10003D) were combined. The protein A/G bead mix was washed with 500 μl ChIP dilution buffer (with 0.15% SDS and 0.1% BSA), moved to a magnetic rack for 1 min and the supernatant was discarded. The wash step was repeated twice. Protein A/G beads were dissolved in 20 μl ChIP dilution buffer (with 0.15% SDS and 0.1% BSA) per IP sample. Then 20 μL washed protein A/G beads was added to each chromatin-antibody mix and incubated for 1 h at 4°C under constant rotation. Tubes were transferred to a magnetic rack and incubated for 5 min and the supernatant was discarded. The beads were washed for 5 min at 4°C with 400 μl buffer in the following order; 1x with ChIP wash buffer 1 (2 mM EDTA, 20 mM Tris (pH 8), 1% Triton X-100, 0.1% SDS, 150 mM NaCl), 2x with ChIP wash buffer 2 (2 mM EDTA, 20 mM Tris (pH 8), 1% Triton X-100, 0.1% SDS, 500 mM NaCl), 2x with ChIP wash buffer 3 (1 mM EDTA, 10 mM Tris (pH 8)). After washing, chromatin was eluted by adding 200 μL freshly prepared ChIP elution buffer (1% SDS, 0.1 M NaHCO3) per sample and incubation of 20 min at room temperature. Beads were spun shortly and moved to a magnetic rack for 1 min, then the eluate was collected. For decrosslinking 8 μL 5 M NaCl and 2 μL 10 mg/mL proteinase K (NEB, cat: P8107S) was added to each eluate and incubated for at least 4 h (alternatively overnight) at 65°C and constant shaking at 1000 rpm. In parallel, to 33.33 μL of the earlier collected negative chromatin fraction 366.67 μL elution buffer, 16 μL 5M NaCl and 4 μL 10 mg/mL proteinase K (NEB, cat: P8107S) was added and incubated in the same way as chromatin with AB. Fragmented DNA was subsequently purified using the QIAquick MinElute PCR Purification Kit (Qiagen; cat. no. 28006) according to the manufacturer’s instructions, with the following modifications: 40 μL (instead of 10 μL) 3M NaAc pH 5.0 was added to the buffer PB and instead of eluting in 10 μL we eluted the samples in 50 μL Buffer EB (10 mM Tris-Cl (pH 8.5)). The eluate concentration was quantified with the Qubit dsDNA HS kit (Thermo scientific, Q33230) to determine the adapter concentration.

For the library construction we used the Kapa HyperPrep kit (Roche, cat: KK8502). To 50 μL fragmented DNA eluate we added 7 μL End repair & A-tailing buffer and 3 μL End Repair & A-Tailing Enzyme Mix and incubated samples 20°C for 30 min and 65°C for 30 min in a thermo cycler. Subsequently, adapters were ligated using the KAPA Unique Dual-Indexed Adapters Kit (15 μM; Roche, KK8726). For input DNA amounts of 2–5 ng, a final adapter concentration of 28 nM was used, whereas for input DNA amounts below 2 ng, a final adapter concentration of 14 nM was used. For the adapter ligation mix we used 60 μl End repair & A-tailing mix and added 5 μL PCR-grade water, 30 μL ligation buffer, 10 μL DNA ligase and 5 μL of adapter stock. The adapter ligation mix was incubated at 20°C for 15 min in a thermo-mixer. Post-ligation cleanup was performed using a 0.8x ratio of SPRI beads from Agencourt AMPure XP reagent (Beckman Coulter; cat. no. A63880), according to the manufacturer’s instructions. Following elution, 20 μL of product was recovered in elution buffer (10 mM Tris-HCl, pH 8.0). For the library amplification we used 20 μL of the adapter-ligated library, 25 μL 2x KAPA HiFi Hotstart Ready-mix and 5 μL 10x Library Amplification Primer Mix. Thermal cycling was conducted with an initial denaturation at 98°C for 45 s, followed by 10 cycles of 98°C for 15 s, 60°C for 30 s, and 72°C for 30 s, with a final extension at 72°C for 1 min. Libraries were stored at 4°C or −20°C for up to 72 h before the post-amplification cleanup. Finally, samples were purified using the Qiaquick MinElute PCR Purification kit (Qiagen, cat: 28006) according to the manufacturer’s instructions with the following modifications: After adding the eluate to the column it was heated to 37°C for 1 min before centrifugation. Samples were eluted in 20 μL Buffer EB (10 mM Tris-Cl, pH 8.5). After the library construction, samples were PAGE-purified, according to the protocol described by.^43^ Quantification of libraries was done on the Agilent tapestation 2200 bioanalyzer, and libraries were equimolarly pooled. Sequencing was done on the DNBseq platform using Paired-End 100 sequencing.

#### Analysis of ChIP-seq data

ChIP-seq data was aligned to the hg38 assembly of the human genome using Bowtie v1.3.1 (parameters: -l 28 -n 2 -–1 --b–t --str–a --chunkmbs 3200),^37^ retaining only uniquely aligned reads. Correlation, principal component analysis, heatmap generation were performed using deepTools v3.5.0.^38^

For peak-level analysis, we segmented the genome into 1,000 bp-long bins, and calculated the number of reads overlapping each bin across each ChIP-seq experiment. After removing all bins with 0 counts across all experiments, we generated a count table that was then processed using DESeq2,^39^ hence taking advantage of replicate analysis. We then identified significantly enriched peaks as those having adjusted *p*-value <0.05 and log2 fold-change ≥ 1.

#### RNA-seq

PBMC-differentiated macrophages were seeded in 6-well plate at 1,000,000 cells/well, cells were treated with 1 μg/mL LPS, 20 ng/mL IFN-γ and 0.1 mM, 1 mM or 10 mM sodium butyrate for 24 h. Samples were washed twice in PBS and lysed by direct addition of 1 mL TRIzol reagent (ThermoFisher Scientific, 15596026). RNA extraction was performed as per manufacturer instructions, and RNA integrity was assessed by electrophoresis. Library preparation was performed using the NEBNext Ultra II RNA Library Prep Kit for Illumina (New England Biolabs, E7770L), as per manufacturer instructions. Libraries were sequenced on an Illumina NextSeq 1000 sequencer.

RNA-seq data was aligned to the hg38 assembly of the human genome using STAR v2.7.10b (parameters: --outFilterMultimapNmax 20 --alignSJoverhangMin 8 --alignSJDBoverhangMin 1 --outFilterMismatchNmax 999 --outFilterMismatchNoverReadLmax 0.04 --alignIntronMin 20 --alignIntronMax 1000000 --alignMatesGapMax 1000000).^40^ Gene counts were computed using featureCounts v2.0.0 and the latest refFlat annotation from UCSC (parameters: -M -p). Differential expression analysis was performed using DESeq2 v1.40.1. Pathway enrichment analysis was performed using the package pathfindR v2.2.0.

#### Western blotting

1×10^6^ human peripheral blood mononuclear-derived macrophages per condition were treated with 1 μg/mL LPS, 20 ng/mL IFN-γ, 0.1 mM, 1 mM or 10 mM sodium butyrate, 10 μM Trichostatin A (TSA) (Sigma, T1952)), 10 μM A485 (Selleck Chemicals, S8740), 10 μM RGFP966 (Sigma), and 10 μM TMP195 (Selleck Chemicals) for 24 h. Then, cells were scraped and lysed using RIPA Lysis and Extraction Buffer (Thermo Scientific) with Protease inhibitor (Roche) and PhosSTOP (Roche). Although RIPA-based extraction may not release all histones from chromatin, published protocols indicate that SDS-based extraction methods similar to RIPA can be appropriate for low-input samples (0.5–5 × 10^6^ cells).^44^ Importantly, in our study the same extraction procedure was applied consistently across all conditions allowing for comparative conclusions between conditions.

Lysates were quantified using Micro BCA Protein Assay Kit (Thermo Scientific). The same amount of lysate of each condition was loaded and run on 4–20% Mini-Protein TGX precast gels (Bio-Rad) at 100 V, and then blotted to PVDF membranes at 90 V, 60 min. Blots were blocked in 5% BSA (Fisher Scientific) at room temperature for 1 h. After blocking, blots were washed three times in 0.01% TBST and then probed with polyclonal rabbit H3AcK27 antibody (Diagenode, C15410196) at 1:1000, polyclonal rabbit H3AcK4+9 + 14+18 + 23+27 (Ac-H3Kn) antibody at 1:1000 (Abcam, ab300641), monoclonal mouse Histone H3 antibody at 1:100 (Santa Cruz, sc-517576), polyclonal rabbit H4AcK5+8 + 12+16 (Ac-H4Kn) antibody at 1:1000 (Abcam, ab177790), anti-Acetyl-EP300-Lys1542 antibody at 1:500 (St ’John’s Laboratory, STJ97696), or monoclonal mouse GAPDH antibody at 1:1000 (Santa Cruz, sc-365062) at 4 °C overnight. The next day, blots were washed three times in 0.01% TBST and then labeled with Donkey anti Rabbit IgG IRDye680 (LI-COR, 926–32223) or Donkey anti Mouse IgG IRDye680 (LI-COR, 926–32222) both at 1:5000 at room temperature for 1 h. After three times washing the blots were scanned using the Odyssey CLx Infrared Imaging System and analyzed using ImageStudio (LI-COR). Blots from the immunoprecipitation of EGFP-p300 were also probed with Ac-H3Kn antibody (Abcam, ab300641) and GAPDH antibody (Santa Cruz, sc-365062) both at 1:1000 and monoclonal mouse GFP (Roche, 11814460001) both at 1:500.

#### Confocal microscopy

50,000 differentiated human macrophages per well were plated on 12-mm diameter glass coverslips and treated with 1 μg/mL LPS, 20 ng/mL IFN-γ, 0.1 mM, 1 mM or 10 mM sodium butyrate, 10 μM Trichostatin A (TSA; Sigma, T1952), and 10 μM Thiomyristoyl (Selleck Chemicals, S8245) for 24 h. Cells were washed three times in PBS and fixed in pre-chilled methanol at −20°C for 10 min and blocked in PBS (Gibco) with 2% human serum (Sigma) at 4 °C for 1 h followed by three times washing. Cells were incubated with primary antibody Ac-H3Kn (Abcam, ab300641) and Human Lamin A/C (BD, 612163) both at 1:500, or with Anti-HP1α alpha antibody Abcam, 3A11F8) at 1:100, in the blocking buffer at 4 °C overnight. The next day, cells were washed in PBS three times and coverslips were incubated for 1 h at room temperature with the combination of the secondary antibodies Donkey anti-Rabbit IgG (H + L) Alexa Fluor 647 (Invitrogen, A31573) and Donkey anti-Mouse IgG (H + L) Alexa Fluor 488 (Invitrogen, A21202) both at 1:400. The coverslips were mounted in 70% glycerol with DAPI. Imaging was performed on a LSM800 Zeiss microscope with a 63× oil lens. Microscope images were analyzed using FIJI ImageJ.^41^ Pearson correlation coefficients (PCC) were calculated automatically using a macro in ImageJ (macro provided in Supplementary Information).

#### Transfection and overexpression

1×10^7^ HeLa cells were plated in 10 mL DMEM medium in 75 cm^2^ cell culture flasks (Corning). eGFP-p300, eGFP-p300 (D1399Y), and eGFP-p300 (N1196D and R1234E) plasmids were transfected into HeLa cells using jetPEI DNA transfection reagent (Polyplus, 101000053). eGFP-p300 and eGFP-p300 (D1399Y) plasmids were gifts from Daniel Gerlich (Addgene plasmids #191764; #191763).^31^ eGFP-p300 (N1196D and R1234E) was generated from eGFP-p300 by point mutations. The plasmid has been deposited to Addgene (#258517). A mixture of 40 μg of plasmids and 160 μL jetPEI reagent was added to 1×10^7^ HeLa cells and then incubated at 37°C, 5% CO_2_ for 24 h.

#### Immunoprecipitation

HeLa cells were treated with 10 mM sodium butyrate, 10 μM A485 and 10 or 50 μM I-CBP112 (Sigma, SML1134) for 24 h, then cells were scraped and lysed using lysis buffer containing 25 mM Tris-HCl, 1% Triton X-100, 1 mM EDTA, 5% glycerol, and 150 mM NaCl, with Protease inhibitor and PhosSTOP. Lysates were quantified using the Micro BCA Protein Assay Kit (Thermo Scientific). 1 mg protein was combined with 2.4 μg polyclonal rabbit GFP antibody (Rockland, 600-401-215) or polyclonal rabbit H3AcK4+9 + 14+18 + 23+27 (Ac-H3Kn) antibody at 1:1000 (Abcam, ab300641). The reaction volume was brought to 500 μL with the cell’s lysis buffer and incubated for 2 h at room temperature. Subsequently, 60 μg washed Pierce Protein A/G Magnetic Beads (Thermo Scientific) were added per 2.4 μg GFP antibody. The mixture was incubated for 1 h at room temperature. The beads were collected using a DynaMag magnets stand (Thermo Scientific) and washed in 0.05% TBST for three times. Finally, the beads were washed in UltraPure Distilled Water (Invitrogen), resuspended in 100 μL SDS-PAGE reducing sample buffer (Bio-Rad) and heated at 95°C for 10 min.

#### Molecular dynamics simulations

The MD (Molecular Dynamics) simulations in this study were performed with GROMACS^42^ involve four different protein systems related to the p300 acetyltransferase: Non-acetylated p300 based on structure PDB 6GYR^30^; Bromodomain, RING-loop and PHD-domains of non-acetylated p300 is based on structure PDB 6GYR, including residues 1045–1290; Bromodomain, RING-loop, and PHD-domain of p300 with three acetylated lysines based on structure PDB 6GYR with lysine residues 1180, 1203, 1228 converted to acetylated lysine; Bromodomain, RING-loop, and PHD-domain of p300 in contact with the histone protein of the nucleosome is based on structure PDB 8HAG.^27^

6GYR is a tetrameric structure; we have considered a single monomer. Additionally, the missing residues 1534–1565 from the HAT were reconstructed using MODELLER.^45^ To speed up the simulations, since we are interested in studying the interaction between the bromodomain and RING-loop, we built a smaller model comprising of the Bromodomain, RING-loop, and PHD-domains covering residues 1045–1290. Here, besides the non-acetylated form, we considered the triple acetylated form K1180Ac, K1203Ac, and K1128Ac where the three lysines in the RING-loop were converted to acetylated lysines. The parameters for lysine and the acetyl group are available in common force fields; however, a set of parameters (charges) specifically designed for the neutral group of acetylated lysine was missing. Therefore, we parameterized them to be compatible with the amber force field. We performed a QM calculation with the Gaussian16 program^46^ to generate the electrostatic potential, which was then used for deriving the RESP charges of the acetylated lysine (ACL) residues via Antechamber.^47^ After manually transformed the 1180, 1203 and 1230 residue from lysine to acetylated lysine, the structures were processed by the GROMACS protein builder gmx pdb2gmx using the all-atom AMBER model amber99sb-ildn.^48^ A residue for the acetylated lysine was added to the library for the builder (abbreviated as ACL) with the new parameters.

To study the interaction between the bromo-domain and the peptide chain of the histone protein, we constructed a reduced model based on the PDB 8HAG structure.^27^ This structure contains the entire P300 acetyltransferase in contact with the nucleosome. Thus, as done previously, we kept only the bromodomain, RING-loop, and PHD-domain of the enzyme. Meanwhile, we kept only the first hundred residues of the final peptide chain (in contact with the bromodomain) of the histone protein of the nucleosome. This latter contains ACL residues, so here too we used the acetylated lysine parameters parameterized earlier.

In the structure of p300 bound to the nucleosome (PDB: 8HAG),^27^ the ten N-terminal residues of H4 remain unresolved and was reconstructed using MODELLER. Specifically, the target sequence was aligned to the 8HAG.pdb template structure, and our custom script generates three candidate models, focusing on refining the N-terminal based on the sequence alignment. An MD-based loop refinement method was applied to further optimize the N-terminal conformations. After generating the models, we assessed their quality using two established scoring methods: DOPE (Discrete Optimized Protein Energy) for evaluating the model’s energy and geometry, and GA341, which estimates the reliability of the model. The structure with the lowest DOPE score (more negative) (DOPE Score = −66243) and a GA341 score of 1 was selected as the most reliable model.

The resulting protein system was solvated in TIP3P water molecules, adding Na^+^ and Cl^−^ ions to maintain neutrality and a salt concentration of 0.15 M. The amber99sb-ildn force field was used.^48^ The velocity rescale thermostat^49^ was used to maintain the temperature at 300 K and an isotropic Parrinello–Rahman barostat^50^ with a reference pressure of 1 atm and isothermal compressibility of 4.5 × 10^−5^ bar^−1^ was used to maintain the pressure of the system. Long-range interactions were evaluated using particle-mesh Ewald summation.^51^ Following minimization and equilibration steps, the systems were simulated for 1 μs, using a time step of 2 fs. All the simulations were performed using the GROMACS-2021.5 package.^52^

### Quantification and statistical analysis

Statistical analyses were performed using GraphPad software. Comparisons between two groups were conducted using two-sided Student’s t-tests, while comparisons among multiple groups were analyzed using ANOVA followed by appropriate post hoc tests. The specific statistical tests used and sample sizes are indicated in the figure legends. *p* values below 0.05 were considered statistically significant, as indicated in the figure legends.
