# Supplementary material for: Butyrate causes loss of specific acetylation by the histone acetyltransferase p300
Source: iScience. 2026 Jul 8;29(7):116528. doi: 10.1016/j.isci.2026.116528 (PMC13378256; doi:10.1016/j.isci.2026.116528)
Supplement: Document S1. Figures S1–S11, Table S1, and Data S1 and S2 [file mmc1.pdf]

iScience, Volume *29*

Supplemental information

Butyrate causes loss of specific acetylation

by the histone acetyltransferase p300

Muwei Jiang, Anthi Psoma, Jinxiao Lyu, Maria Gabriella Chiariello, Rutger Modderman, Frans Bianchi, Danny Incarnato, Siewert-Jan Marrink, Iris H. Jonkers, and Geert van den Bogaart

Contents:

- Supplementary Figures 1–11
- Supplementary Table 1: primers for RT-qPCR
- Supplementary Data 1: FIJI macro for quantification of intensity
- Supplementary Data 2: FIJI macro for calculation of the Pearson correlation coefficient

Supplementary Fig. 1

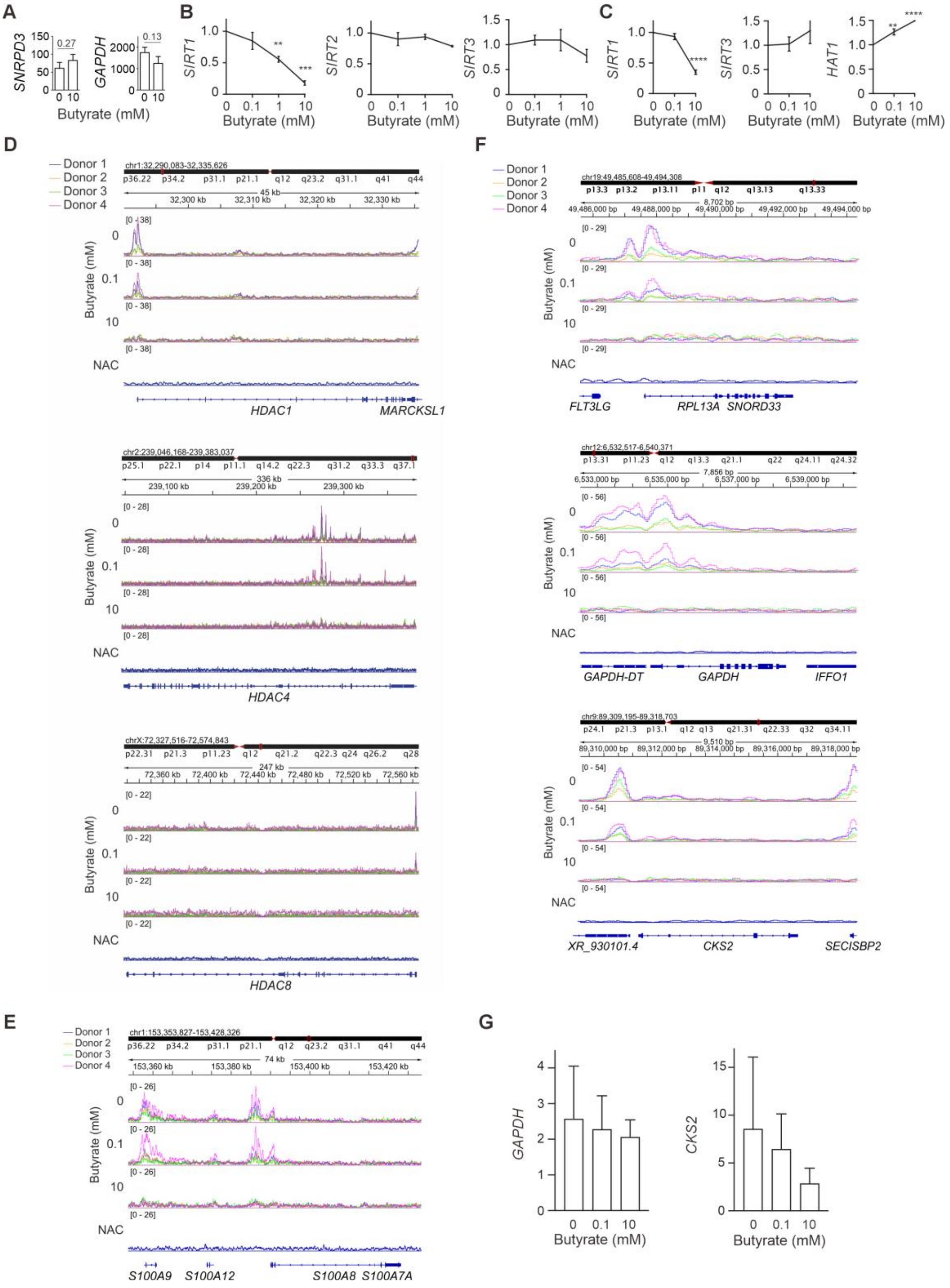

Supplementary Figure 1. RT-qPCR and ChIP-Seq of H3K27Ac. Human peripheral blood monocyte-derived macrophages were stimulated for 24 hr with LPS, IFN-γ and the indicated concentrations of Na-butyrate. (**A**) *SNRPD3* and *GAPDH* mRNA levels by RNA-seq normalized to human genome (paired 2-sided t-test, n = 4 donors; error

bars represent means ± SEM) (**B**) *SIRT1*, *SIRT2*, and *SIRT3* mRNA levels by RT-qPCR normalized to without butyrate. (One-way ANOVA with a Dunnett's multiple comparisons test, n=3 donors; error bars represent means ± SEM; **: P < 0.01; ***: P < 0.001; conditions were compared with the no butyrate condition) (**C**) *SIRT1*, *SIRT3*, and *HAT1* mRNA levels by RNA-seq normalized to human genome (One-way ANOVA with a Dunnett's multiple comparisons test; n=4 donors; error bars represent means ± SEM; ****: P < 0.0001). (**D**-**F**) Genome browser snapshots of published ChIP-seq data[1] showing H3K27Ac enrichment in (**D**) *HDAC1, HDAC8, HDAC4,* (**E**) *S100A9, S100A8*, (**F**) *RPL13A, GAPDH,* and flanking genes. NAC: no antibody control. (**G**) *GAPDH* and *CKS2* mRNA levels by ChIP-qPCR normalized to genomic DNA. (n = 4 donors; error bars represent means ± SEM)

Supplementary Fig. 2

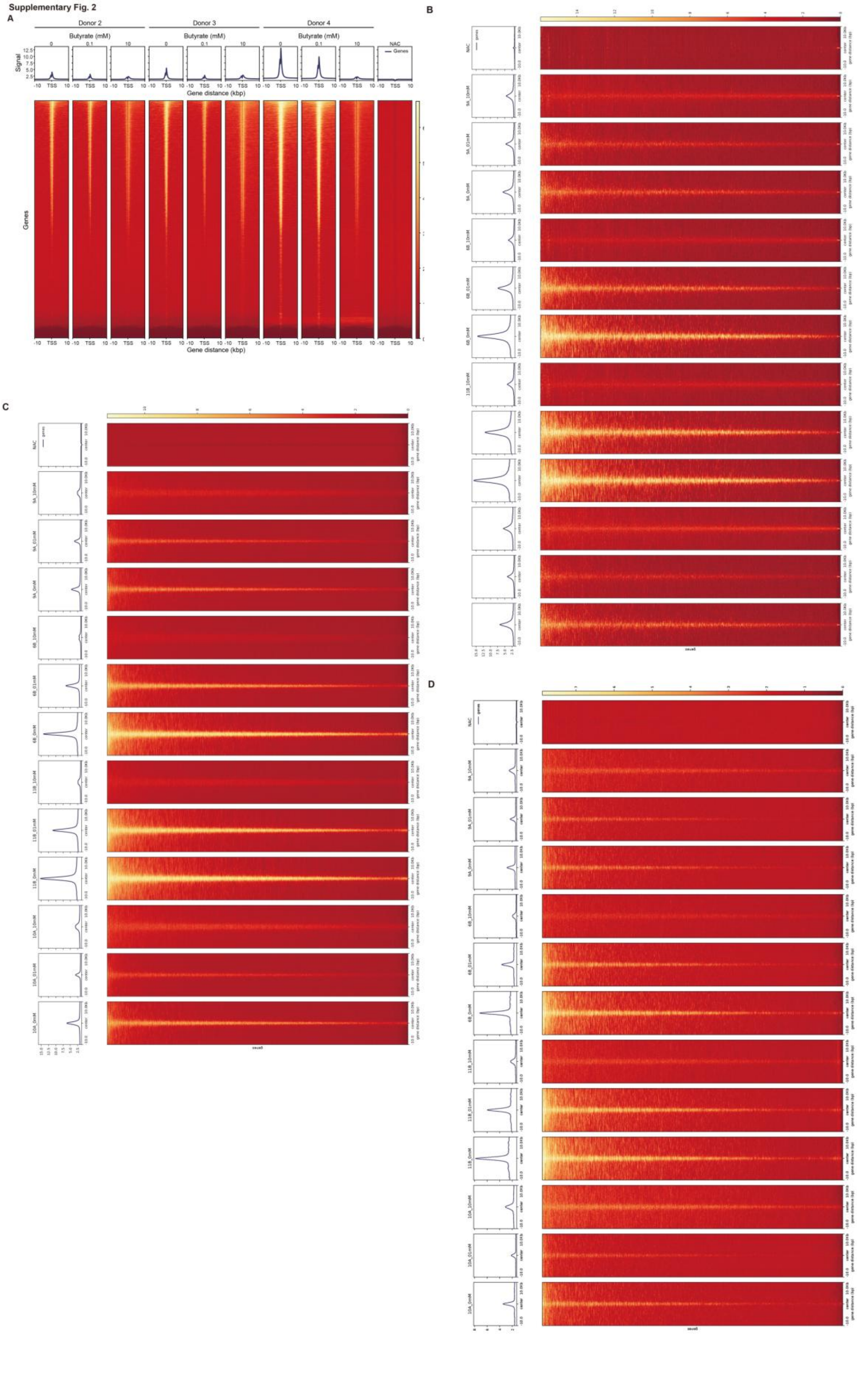

Supplementary Figure 2. Butyrate decreases histone acetylation at specific sites. (**A**) Intensity profile overlaps from published ChIP-seq data[1] of all transcription start sites (TSS) for donors 2–3 (first donor shown in main Fig. 1C), showing a dose-dependent decrease in fold-enrichment of peaks relative to total reads. (**B**) Heatmap displays the subset of peaks, identified by peak calling of H3K27ac (using MACS 1.4.2), which are common to all 4 individuals at no butyrate condition. (**C**) Heatmap displays regions identified as significantly changing their acetylation level between 0 mM and 10 mM butyrate. These were identified by binning the genome using a sliding window of 1000 bp and by then using DeSeq2 (R) to identify regions of significant change in acetyl level ($|\log2(\text{Fold-change})| >= 1$, adjusted p-value < 0.05). (**D**) Heatmap displays regions corresponding to active enhancers in CD14-positive monocytes using ENCODE data. These were identified by selecting genomic regions that were H3K4me1 and H3K27ac positive, but H3K4me3 negative. The procedure was to first call peaks for H3K4me1, H3K4me3 and H3K27ac ChIP-seqs (using MACS 1.4.2), and then, using the intersect utility from BEDtools v2.31.0, overlapping H3K4me1 and H3K27ac peaks were selected, and any peak overlapping with a H3K4me3 peak was discarded.

Supplementary Fig. 3

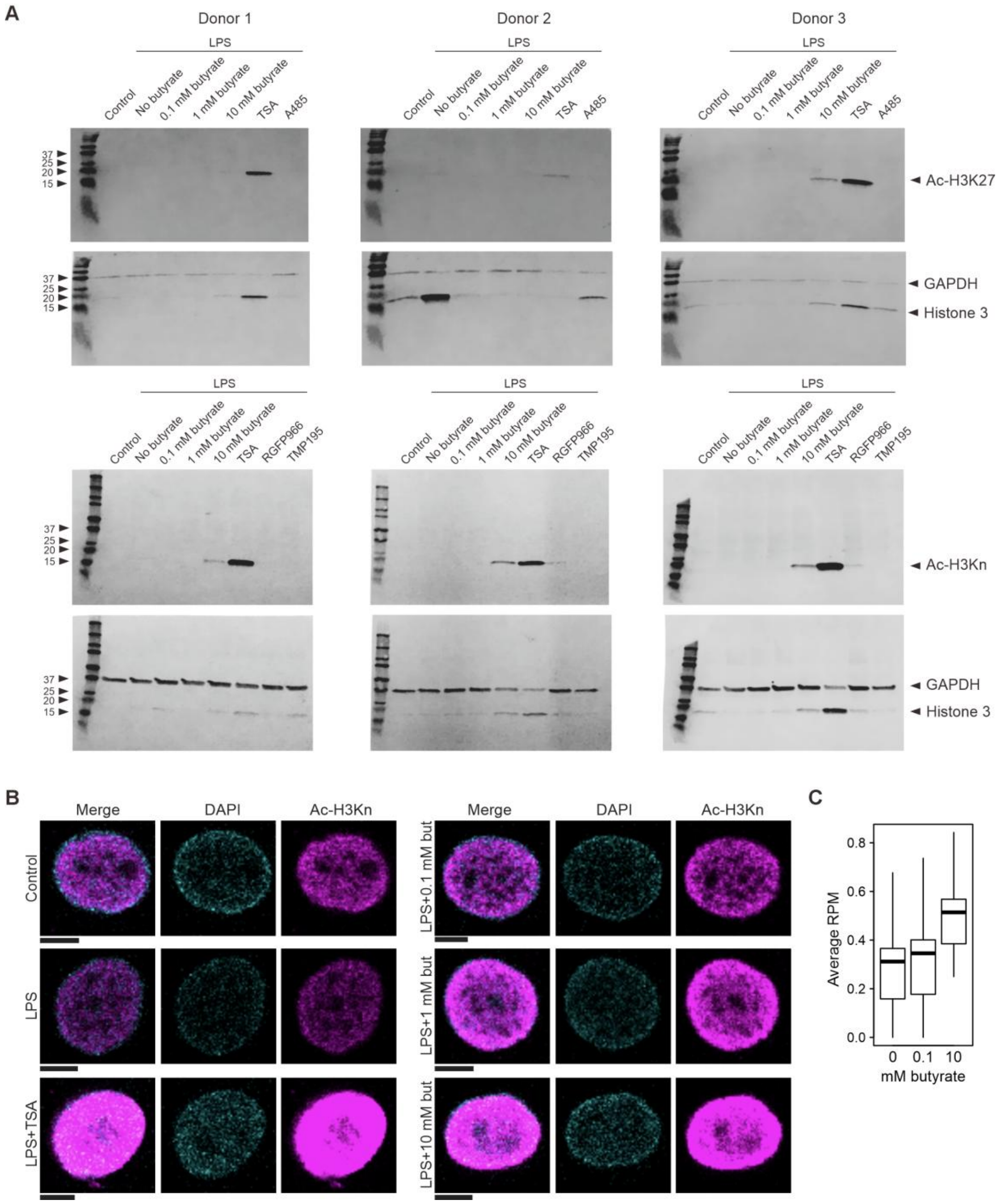

Supplementary Figure 3. Butyrate induces hyperacetylation of histones globally. Human peripheral blood monocyte-derived macrophages were stimulated for 24 hr with LPS, IFN-γ, the indicated concentrations of Na-butyrate, 10 μM HDAC inhibitor TSA, RGFP966, TMP195, and/or 10 μM HAT inhibitor A485. (**A**) Complete Western blots for 3 donors showing histone acetylation detected with two different antibodies recognizing acetylated (Ac-)H3K27 and H3K4+9+14+18+23+27 (Ac-H3Kn). Blots were stripped and reprobed for GAPDH (loading control) and total H3, however, the

total H3 showed poor staining possibly due to incomplete stripping. Prolonged strpping resulted in overstripping, whereas shorter stripping times led to substantial residual background. (**B**) Representative confocal images from 3 donors for histone acetylation (Ac-H3Kn; magenta) and DAPI (cyan) (at least 5 cells per donor). Some pictures of the Ac-H3Kn staining are saturated to highlight the increase in staining intensity. Scale bars: 5 μm. (**C**) Background increase in H3K27Ac signal by ChIP-seq. 1,000,000 genomic regions located at least 5 kb away from any annotated gene were randomly selected and the normalized signal within each region was calculated.

Supplementary Fig. 4

A B

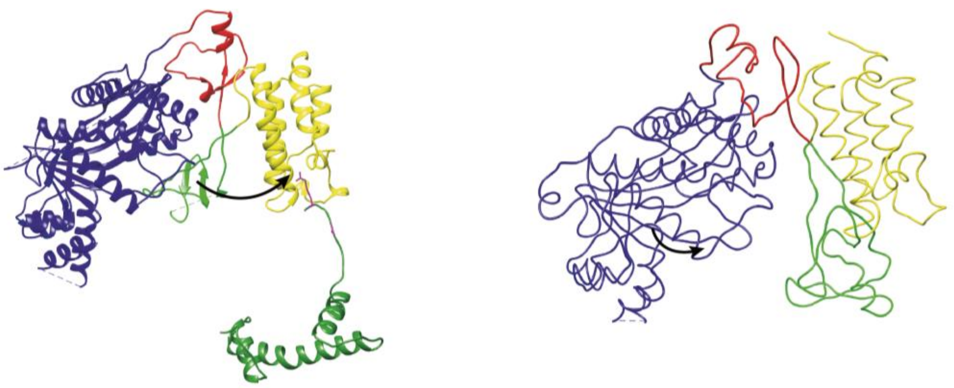

Supplementary Figure 4. Comparison of cryo-EM structures suggests that the RING loop moves between the HAT and bromodomain. Different protein sub-units are color-coded: bromodomain (Bd; yellow), RING-loop (green), PHD-domain (red), HAT-domain (blue), and histone H4 (dark green). (**A**) Cryo-EM model of p300, with its Bd bound to the acetylated histone 4 (H4) of the H4K12acK16ac nucleosome (pdb code 8HAG)[3]. For clarity, only p300 and acetylated H4 is shown. (**B**) Low resolution cryo-EM structure of p300 without substrate (pdb code: 6K4N)[4]. Note that the RING loop is tilted towards the binding pocked of the Bd (arrows).

Supplementary Fig. 5

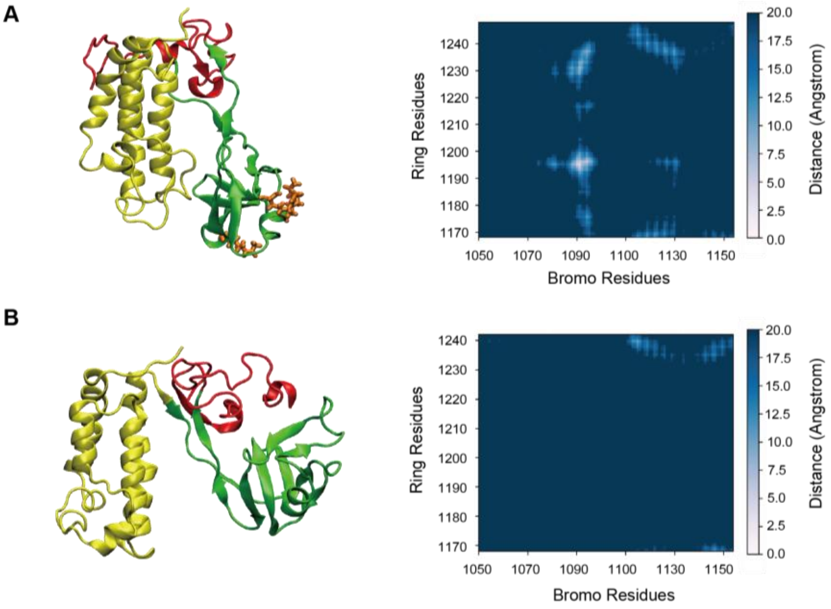

Supplementary Figure 5. MD simulations of the p300 acetyltransferase system. The reference structure is PDB 6GYR. Different protein sub-units are color-coded: bromodomain (yellow), RING-loop (green), and PHD-domain (red). Structure extracted from the simulation of the triple acetylated form K1180Ac, K1203Ac, and K1228Ac, with acetylated lysines highlighted in orange (**A**) and the non-acetylated form (**B**). The contact maps between the bromodomain and RING-loop residues are shown in the right panel for both the systems.

Supplementary Fig. 6

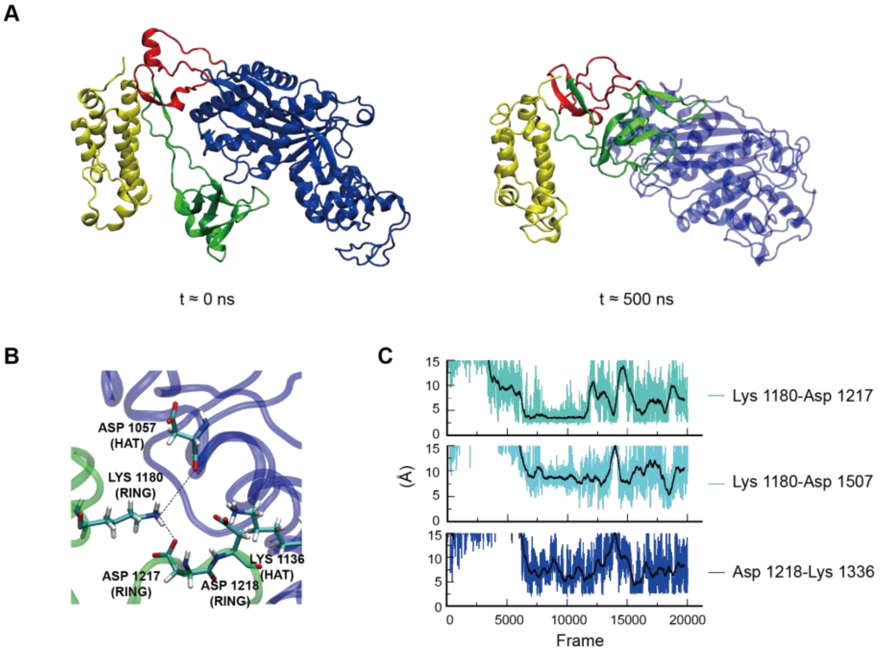

Supplementary Figure 6. MD simulations of the full non-acetylated p300 acetyltransferase system. Different protein sub-units are color-coded: bromodomain (yellow), RING-loop (green), PHD-domain (red) and HAT (blue). (**A**) Representative structures at time 0 and 500 ns simulated time. (**B**) The RING-loop is attached to the HAT through a network of interactions involving K1180. (**C**) H-bond interactions between the RING-loop and HAT over the simulation time.

Supplementary Fig. 7

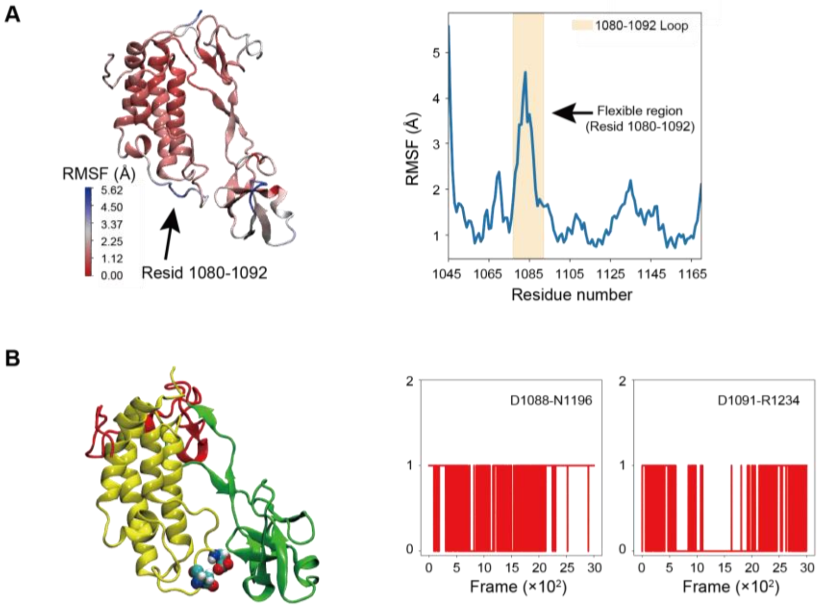

Supplementary Figure 7. MD simulations of the interactions between the bromodomain and RING-loop. (**A**) Root mean square fluctuations (RMSF) of the bromodomain (residue number 1045–1168). The structure colored according to the RMSF value is also shown. (**B**) Number of contacts between D1088 and D1091 (bromodomain) and N1196 and R1234 (RING-loop) over the simulation time. Representative structure with the D1088 and D1091 in ball-and-stick representation located on the flexible loop of the bromodomain.

Supplementary Fig. 8

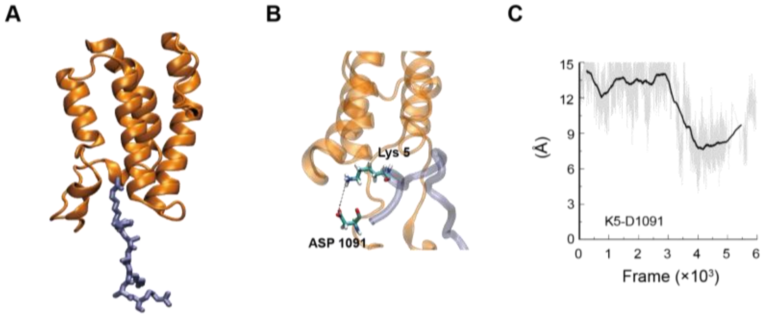

Supplementary Figure 8. MD simulations of the p300 acetyltransferase system bound to acetylated histone H4. The reference structure is PDB 8HAG. Different protein sub-units are color-coded: bromodomain (orange), histone H4 N-terminal peptide chain (ice blue). (**A**) Histone chain buried into the bromo domain from the cryo EM structure 8HAG. Here the residues 1–10 are missing. (**B**) Snapshot from MD simulation, where the missing residues 1–10 were reconstructed. The interaction between K5 and D1091 is highlighted. (**C**) The distance between K5 and D1091 over the simulation frames is shown.

Supplementary Fig. 9

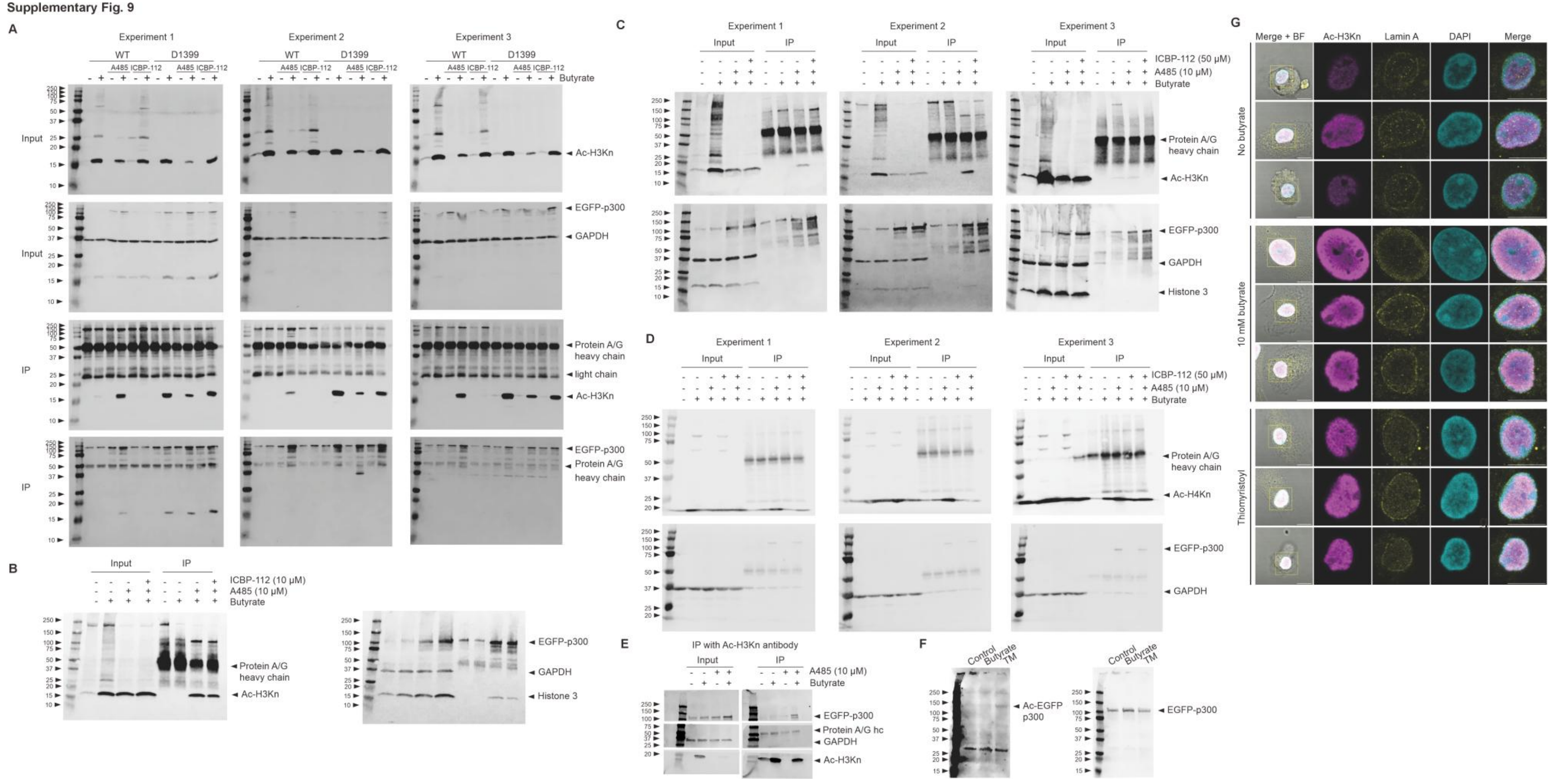

Supplementary Figure 9. Butyrylated p300 does not bind to acetylated H3. (**A**) HeLa cells were transfected with EGFP-tagged catalytic core of p300 wildtype or catalytically inactive D1399Y mutant and stimulated for 24 hr with 10 mM Na-butyrate, 10 μM HAT inhibitor, and/or 10 μM bromodomain inhibitor prior to IP for EGFP. Complete Western blots for 3 independent experiments, showing Ac-H3K4+9+14+18+23+27 (Ac-H3Kn), EGFP-p300, and GAPDH detected in lysates and IP samples. (**B-C**) Transfected Hela cells were stimulated for 24 hr with 10 mM Na-butyrate, 10 μM A485, and 10 μM (**B**) or 50 μM (**C**) ICBP-112 prior to IP with an antibody against EGFP. Total H3 showed poor staining possibly due to incomplete stripping. Prolonged strpping resulted in overstripping, whereas shorter stripping times led to substantial residual background. (**D**) Transfected Hela cells were stimulated for 24 hr with 10 mM Na-butyrate, 10 μM A485, and 50 μM ICBP-112 prior to IP with an antibody against EGFP. Western blot showing Ac-H4K5+8+12+16 (Ac-H4Kn) in lysates and IP samples. (**E**) Reciprocal IP as panel A, using IP with antibody against Ac-H3Kn. (**F**) Transfected Hela cells were stimulated for 24 hr with 10 mM Na-butyrate, 10 μM of SIRT2 inhibitor Thiomyristoyl (TM), or 10 μM A485 followed by Western blot with an antibody recognizing an acetylated lysine located in the AIL of p300 (K1542). (**G**) Human peripheral blood monocyte-derived macrophages were stimulated for 24 hr with LPS, IFN-γ, and the indicated concentrations of Na-butyrate or 10 μM TM. Representative confocal images from 3 donors for Ac-H3Kn (magenta) (at least 5 cells per donor), Lamin A (yellow), and DAPI (cyan). Scale bars: 5 μm.

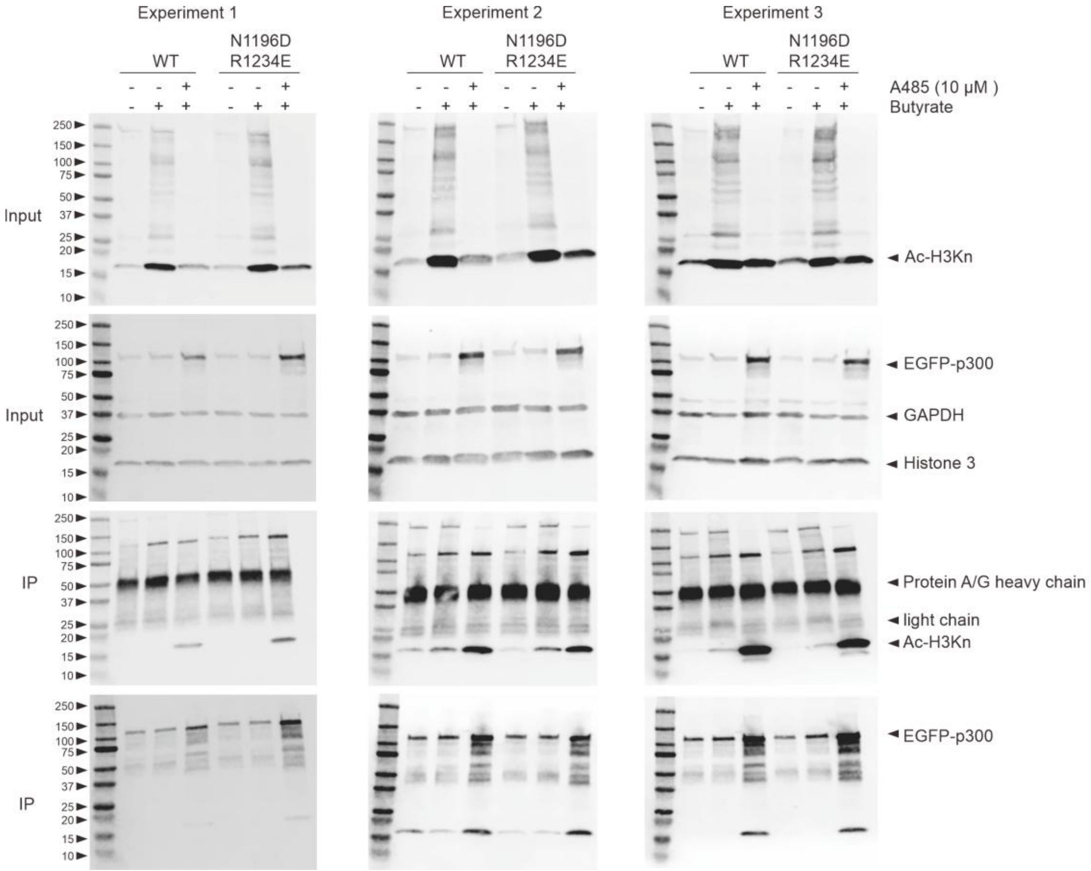

Supplementary Figure 10. N1196 and R1234 of RING loop might suppress p300 bromodomain binding to acetylated histone. Hela cells were transfected with the EGFP tagged catalytic core of p300 wildtype or N1196D and R1234E mutant, and stimulated for 24 hr with 10 mM Na-butyrate, 10 µM HAT inhibitor A485 prior to immunoprecipitation (IP) for EGFP. Western blot showing Ac-H3K4+9+14+18+23+27 (Ac-H3Kn), EGFP-p300, histone 3 (H3) and GAPDH detected in lysates and IP samples. Total H3 showed poor staining possibly due to incomplete stripping. Prolonged strpping resulted in overstripping, whereas shorter stripping times led to substantial residual background.

Supplementary Fig. 11

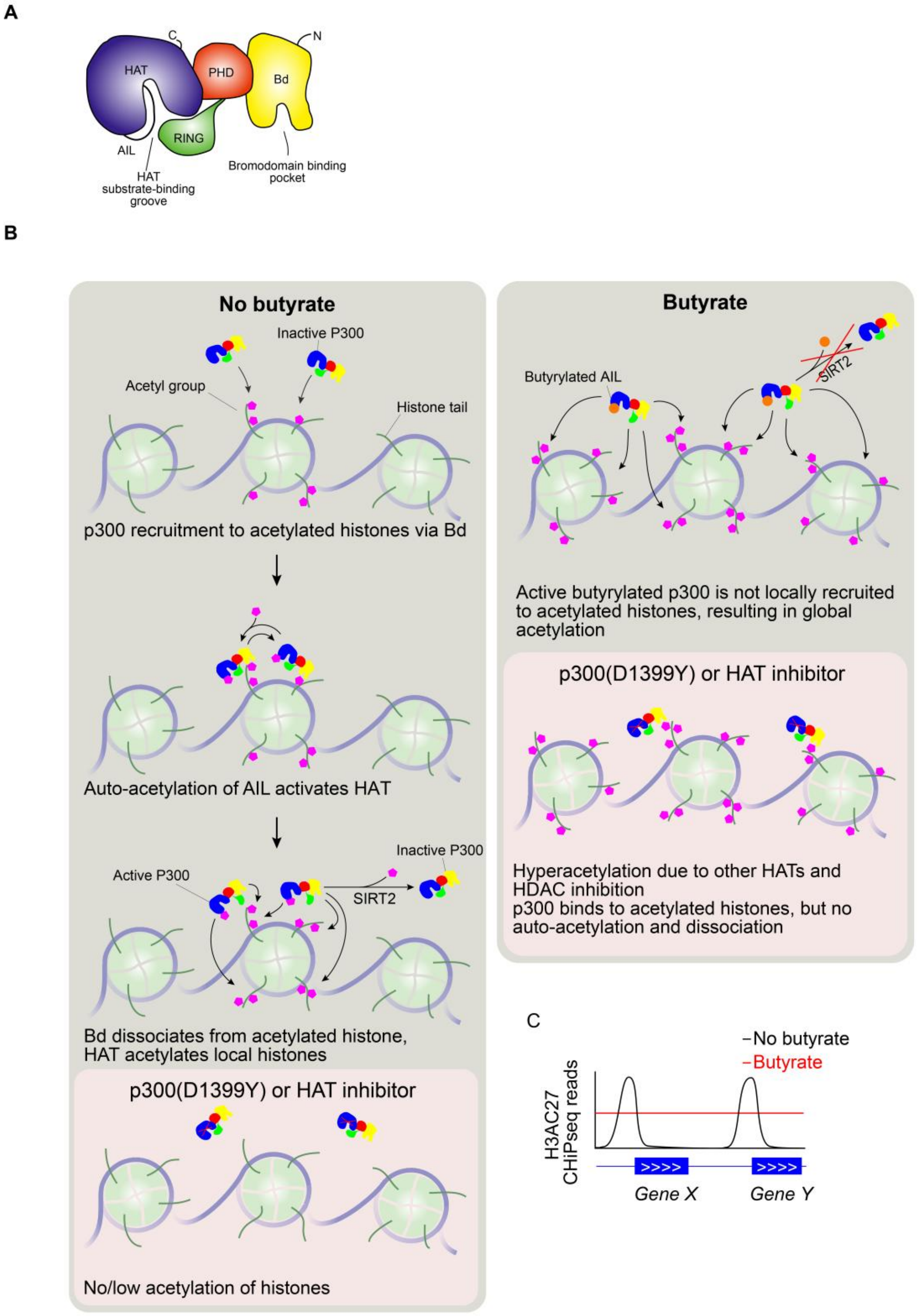

Supplementary Figure 11. Model for loss of specific histone acetylation by butyrate. (**A**) Domain topology of the catalytic core of p300. Yellow: bromodomain (Bd); green: RING-loop: red, PHD domain: blue, HAT domain: AIL: autoinhibitory loop. Adapted from[2]. (**B**) Proposed model for histone acetylation by p300 in the absence and presence

of butyrate. In the absence of butyrate, active p300 molecules are recruited to acetylated histones by binding to its Bd. This promotes in-*trans* auto-acetylation of the AIL, triggering its activation. Lysines located in the RING-loop also become acetylated, weakening the interaction with the HAT. The RING-loop can now interact with the bromodomain and thereby prevent binding to acetylated histones. Activated p300 dissociates from acetylated histones and acetylates other histones nearby. p300 is inactivated by SIRT2, which deacetylates the AIL. Histone acetylation is reduced with the catalytically inactive p300 mutant or with a HAT inhibitor. Butyrate induces butyrylation of the AIL of p300, resulting in its hyperactivation. However, the Bd of butyrylated p300 is not recruited to acetylated histones. In addition, SIRT2 is less effective in removing p300 butyrylation compared to the acetylation. These effects result in non-specific hyperacetylation of histones. Butyrylation of p300 is blocked with a catalytically inactive mutant or with a p300 inhibitor, and the Bd of p300 remains associated to acetylated histones. In these conditions, butyrate still increases histone acetylation due to its inhibition of HDACs and the activities of other HATs. (**C**) Butyrate results in global increase of histone acetylation, and loss of specific histone acetylation.

Supplementary Table 1. Primer sequences for reverse transcription-quantitative PCR.

Gene	Primer sequence (5'-3')
HDAC1	F: GGTCCAAATGCAGGCGATTCCT<br>R: TCGGAGAACTCTTCCTCACAGG
HDAC3	F: GAGTTCTGCTCGCGTTACACAG<br>R: CGTTGACATAGCAGAAGCCAGAG
HDAC4	F: AGGTGAAGCAGGAGCCCATTGA<br>R: GGTAGTTCCTCAGCTGGTGGAT
HDAC7	F: TCCTGGCACAGCGGATGTTTGT<br>R: TGAAGGCGAGGTCAGTGACACT
HDAC8	F: TGTGCTGGAAATCACGCCAAGC<br>R: ACCACTCCTCAGCTCTGGAAAC
SIRT1	F: TAGACACGCTGGAACAGGTTGC<br>R: CTCCTCGTACAGCTTCACAGTC
SIRT2	F: CTGCGGAACTTATTCTCCCAGAC<br>R: CCACCAAACAGATGACTCTGCG
SIRT3	F: CCCTGGAAACTACAAGCCCAAC<br>R: GCAGAGGCAAAGGTTCCATGAG
EP300	F: GATGACCCTTCCCAGCCTCAAA<br>R: GCCAGATGATCTCATGGTGAAGG
SNRPD3	F: GGAAGCTCATTGAAGCAGAGGAC<br>R: CAGAAAGCGGATTTTGCTGCCAC

HDAC, histone deacetylase; SIRT, Sirtuin; SNRPD3, Small Nuclear Ribonucleoprotein D3 Polypeptide.

Supplementary Data 1. FIJI macro for automated quantification of the mean intensity of histone acetylation normalized to DAPI.

```
var bittype = 16;

var directory = File.directory;

var id0 = getImageID();

var name0 = getTitle();

run("Duplicate...", "duplicate channels=1");

if (bittype == 8)

{setMinAndMax(1, 1); //8bit}

else {setMinAndMax(100, 100); //16-bit}

run("Apply LUT");

run("8-bit");

rename("ch1");

var id1 = getImageID();

var name1 = getTitle();

selectImage(id0);

if (bittype == 8)

{run("Duplicate...", "duplicate channels=3");//8-bitsetMinAndMax(1, 1); //8bit}

else {run("Duplicate...", "duplicate channels=4");//16-bitsetMinAndMax(100, 100);
```

```
//16-bit}
run("Apply LUT");
run("8-bit");
rename("ch4");
var id2 = getImageID();
var name2 = getTitle();
imageCalculator("Add create", "ch1","ch4");
selectWindow("Result of ch1");
run("32-bit");
var id3 = getImageID();
setAutoThreshold("Default dark no-reset");
//run("Threshold...");
run("NaN Background");
setMinAndMax(0, 1);
selectImage(id1);
close();
selectImage(id2);
close();
selectImage(id0);
```

```
run("Duplicate...", "duplicate channels=1");
rename("ch1");
var id2 = getImageID();
var name2 = getTitle();
imageCalculator("Multiply create 32-bit", "Result of ch1","ch1");
selectWindow("Result of Result of ch1");
var id5 = getImageID();
run("Divide...", "value=255");
if (bittype == 16)
{run("Divide...", "value=256"); //16-bit}
run("Measure");
selectImage(id3);
close();
selectImage(id2);
close();
selectImage(id0);
close();
selectImage(id5);
close();
```

Supplementary Data 2. FIJI macro for calculation of the Pearson correlation coefficient (PCC) between the Lamin A and H3Ac staining.

```
var ID1 = getImageID();

selectImage(ID1);

run("Duplicate...", "duplicate channels=1");

var ID1_ch1 = getImageID();

var Name1_ch1 = getTitle();

selectImage(ID1);

run("Duplicate...", "duplicate channels=2");

var ID1_ch2 = getImageID();

var Name1_ch2 = getTitle();

run("JACoP ", "imga=[" + Name1_ch1 + "] imgb=[" + Name1_ch2 + "] pearson");

selectImage(ID1_ch1);

close();

selectImage(ID1_ch2);

close();

selectImage(ID1);

close();
```

References

1. Jiang, M., Incarnato, D., Modderman, R., Lazaro, A.A., Jonkers, I.H., Bianchi, F., and van den Bogaart, G. (2025). Low butyrate concentrations exert anti-inflammatory and high concentrations exert pro-inflammatory effects on macrophages. J. Nutr. Biochem. *144*, 109962. https://doi.org/10.1016/j.jnutbio.2025.109962.
2. Ortega, E., Rengachari, S., Ibrahim, Z., Hoghoughi, N., Gaucher, J., Holehouse, A.S., Khochbin, S., and Panne, D. (2018). Transcription factor dimerization activates the p300 acetyltransferase. Nature *562*, 538–544. https://doi.org/10.1038/s41586-018-0621-1.
3. Kikuchi, M., Morita, S., Wakamori, M., Sato, S., Uchikubo-Kamo, T., Suzuki, T., Dohmae, N., Shirouzu, M., and Umehara, T. (2023). Epigenetic mechanisms to propagate histone acetylation by p300/CBP. Nat. Commun. *14*, 4103. https://doi.org/10.1038/s41467-023-39735-4.
4. Ghosh, R., Kaypee, S., Shasmal, M., Kundu, T.K., Roy, S., and Sengupta, J. (2019). Tumor Suppressor p53-Mediated Structural Reorganization of the Transcriptional Coactivator p300. Biochemistry *58*, 3434–3443. https://doi.org/10.1021/acs.biochem.9b00333.
